# The role of cytochrome oxidases in bacterial virulence

**DOI:** 10.1128/iai.00609-24

**Published:** 2026-06-15

**Authors:** Liliana S. McKay, Andrew Camilli, Peggy A. Cotter

**Affiliations:** 1 Department of Microbiology and Immunology, School of Medicine, University of North Carolina—Chapel Hill 318275, Chapel Hill, North Carolina, USA; 2 Department of Molecular Biology and Microbiology, Tufts University School of Medicine 12261, Boston, Massachusetts, USA; University of California at Santa Cruz, Santa Cruz, California, USA

**Keywords:** terminal oxidase, bacterial respiration, cytochrome oxidase

## Abstract

Bacterial physiology plays an important role in virulence. Being a pathogen is energetically expensive, requiring the production of specialized virulence factors and other proteins that enable survival within the host. The most efficient way for bacteria to generate the energy required for virulence is by electron-coupled oxidative phosphorylation, with oxygen as the terminal acceptor, also called aerobic respiration. Cytochrome oxidases are critical components of the electron transport chain used in aerobic respiration. Studies on the role of cytochrome oxidases during infection in clinically relevant bacteria have shown that they are often critical, even in low-oxygen environments, such as the gastrointestinal tract. Cytochrome oxidases—especially *bd*-type, the class most associated with virulence—are thus being explored as possible antimicrobial drug targets. However, the mechanisms underlying the role of cytochrome oxidases, especially non-*bd*-type cytochrome oxidases, during infection remain poorly understood in most organisms. In this review, we examine what is known about the role of cytochrome oxidases during infection, highlighting four pathogens: *Mycobacterium tuberculosis* and uropathogenic *Escherichia coli*, in which the role of *bd*-type cytochrome oxidases during infection has been investigated mechanistically, and *Vibrio cholerae* and *Bordetella bronchiseptica*, in which non-*bd*-type cytochrome oxidases contribute to infection. These examples indicate the need to assess what role cytochrome oxidases play in a wider array of bacterial pathogens.

## INTRODUCTION

Researchers investigating bacterial pathogens have traditionally focused on identifying and characterizing virulence factors. However, as our understanding of the interplay between host and pathogen has deepened, the field has developed a greater appreciation for the role of bacterial physiology in infection. Without the adjustments required to live within the various niches they occupy during infection, bacteria would not survive long enough to cause disease. A major component of this physiological adaptation is generating sufficient energy to synthesize the biomolecules required to survive in each encountered host environment.

Catabolism, or the breakdown of larger molecules into smaller ones, releases energy that can be used to drive cellular processes. To generate ATP, the products generated during catabolism get directed either toward oxidative phosphorylation (i.e., respiration), where electrons are transferred along the electron transport chain (ETC) to generate a proton gradient used to drive ATP synthesis, or substrate-level phosphorylation (i.e., fermentation), where ADP gains a phosphate group directly from a substrate molecule. Respiration generates more ATP per molecule broken down than fermentation and is therefore energetically favorable when terminal electron acceptors are available.

In an era of rising antibiotic resistance, new approaches are needed to treat bacterial infections, and understanding the role of bacterial physiology during infection can reveal opportunities for new treatments. Many antibiotics, including aminoglycosides, rely on respiration and the consequent generation of a proton motive force for uptake into target cells (1). However, some bacterial species can tolerate these antibiotics by entering a metabolically dormant state. By not growing or dividing, these tolerant cells avoid the fatal consequences of antibiotics that target critical biosynthetic pathways, such as cell wall synthesis or DNA replication. These tolerant persister populations, which have been reported in increasing numbers of bacterial species, including *Staphylococcus aureus* (2–6)*, Pseudomonas aeruginosa* (7–11)*, Mycobacterium tuberculosis* (12, 13), and *Escherichia coli* (14–16)*,* are thought to be one of the causes of antibiotic treatment failure and recurrent infections. Recent research on *M. tuberculosis* has revealed that inhibiting components of the ETC—including cytochrome oxidases (COs), the final enzymes in the ETCs used for aerobic respiration—can clear antibiotic-tolerant populations (17). Since COs have been shown to be required during infection with other pathogens, targeting COs is a potential direction for broader antimicrobial drug development. Despite the potential revealed by these initial studies, much remains unclear about the role of COs during infection, particularly at a mechanistic level. In this review, we summarize what is known about the roles bacterial COs play during mammalian infection—especially the subclass of COs, *bd-*type, that are most implicated in infection—and highlight examples of organisms that do and do not rely on *bd*-type COs during infection.

## CYTOCHROME OXIDASES CATALYZE THE FINAL STEP OF ELECTRON TRANSPORT-COUPLED OXIDATIVE PHOSPHORYLATION DURING AEROBIC RESPIRATION

**Fig 1:**
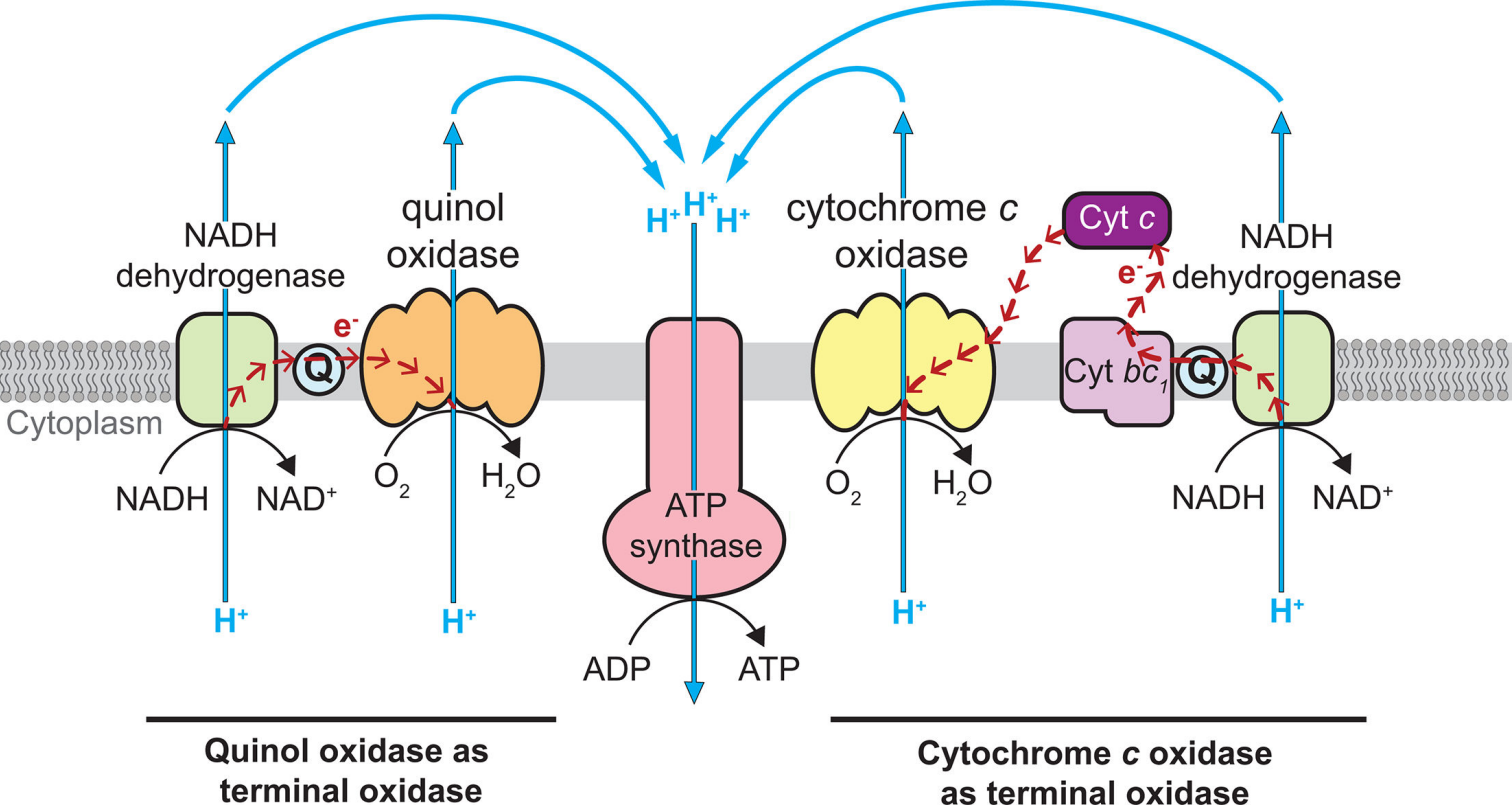
Electron transport-coupled oxidative phosphorylation relies on cytochrome oxidases. Simplified schematic of a theoretical bacterial respiratory chain. Electrons (red dashed line) are shuttled from donors to acceptors with a higher affinity for those electrons. In aerobic respiration, the terminal acceptor is oxygen. The potential differential is used to export protons (cyan line), generating a proton gradient that powers ATP synthase (middle). In respiratory chains using a quinol oxidase as the terminal oxidase (left), electrons are shuttled directly from a quinol (Q) to the cytochrome oxidase. In respiratory chains using a cytochrome *c* oxidase as the terminal oxidase (right), electrons are donated to the cytochrome oxidase from cytochrome *c* (Cyt. *C*)*,* which receives electrons from a quinol via cytochrome *bc_1_* (Cyt. *bc_1_*).

COs, also called terminal oxidases, are a critical component of the ETC used for aerobic respiration. During electron transport-coupled oxidative phosphorylation, electrons are transferred from donors to acceptors that have a higher affinity for those electrons (Fig. 1). The redox potential associated with these reactions is used to move protons across a membrane to generate a proton gradient, which can then be used to power ATP synthase to generate ATP. Unlike eukaryotes, which rely on linear ETCs within mitochondria and chloroplasts, prokaryotes often use branched ETCs involving several terminal oxidases (for aerobic respiration) and/or terminal reductases (for anaerobic respiration). This approach allows bacteria to adapt to their current environments by utilizing the terminal electron acceptors available for respiration (18).

Different types of COs have different functional and structural characteristics that can be used to categorize them. The subtypes of COs are named for the types of hemes (*a, b, c, d,* or *o*) incorporated into the protein complex. *bd-*type COs, as the name implies, utilize heme *b* and heme *d*, while *aa_3_*-type COs, the type used by mitochondria, utilize only heme *a*, with the subscript “3” denoting the heme involved in oxygen reduction (19). Most COs, including those used by eukaryotes, are heme-copper oxidases (HCOs), named for the heme and copper cofactors required for their catalytic activity (20, 21). Bacteria and archaea, however, have a unique class of heme-only COs, called *bd-*type COs (22). The structural and cofactor differences between HCOs and *bd-*type COs contribute to the unique properties of *bd-*type COs, which will be explored further in a following section.

Beyond their cofactors, COs are subclassed by two additional factors: the electron carriers of the ETC with which they interact and their affinity for oxygen. COs can either interact with reduced quinones like ubiquinol, in which case they are called quinol oxidases, or interact with cytochrome *c,* in which case they are called cytochrome *c* oxidases. Having the electron travel to the CO via cytochrome *c* results in a longer ETC with more protons pumped per electron transferred. As for oxygen affinity, COs are labeled as either low or high affinity. Low-affinity COs function optimally at atmospheric oxygen levels, while high-affinity COs can scavenge oxygen more effectively and thus function even under microoxic conditions (23–25). As a consequence of the structural differences associated with increased oxygen affinity, low-affinity COs are more efficient than high-affinity COs, as they generate a greater charge difference across the membrane per electron transferred than high-affinity COs (26). Therefore, while high-affinity COs allow aerobic respiration to continue under conditions when low-affinity COs are nonfunctional, they are less favorable than low-affinity COs under ambient air conditions.

In this review, we highlight four types of COs implicated in bacterial virulence (Table 1). Two are low-affinity COs: *aa_3_*-type, which are cytochrome *c* HCOs, and *bo_3_*-type, which are quinol HCOs. The other two types of COs highlighted are generally high-affinity: *cbb_3_*-type, which are cytochrome *c* HCOs, and *bd*-type, which are quinol oxidases. A subset of *bd-*type COs, called cyanide-insensitive oxidases or CIOs, is low-affinity rather than high-affinity but has the unique feature of higher resistance to potassium cyanide, which is produced by some organisms like *P. aeruginosa* as a consequence of their metabolisms (27–30). By producing COs with different characteristics under different conditions, bacteria can maximize their energy production in the various environments they inhabit.

**TABLE 1:** Common types of cytochrome oxidases and their characteristics

Type	Class	Electron donor	Heme cofactors	Predicted O_2_ affinity
*aa_3_*	HCO	Cytochrome *c*	*a*	Low
*bd*	*bd*-type	Quinol	*b, d*	High^*a*^
*bo_3_*	HCO	Quinol	*b, o*	Low
*cbb_3_*	HCO	Cytochrome *c*	*c, b*	High

^*a*^ A subset of *bd*-type cytochrome oxidases, called cyanide-insensitive oxidases (CIOs), is low affinity.

## CYTOCHROME OXIDASES, ESPECIALLY *BD-*TYPE CYTOCHROME OXIDASES, ARE LINKED TO VIRULENCE AND TISSUE TROPISM IN PATHOGENS

Researchers have found that COs are important for virulence in many clinically relevant pathogens. The results of these studies are summarized in Table 2. While this analysis primarily includes studies using either animal models of infection or human patient samples, screens for factors required for infection have also been included when available and relevant. The majority of studied organisms (82%) relied on *bd-*type COs during infection, either alone or in conjunction with another CO. Of the HCOs, *aa_3_*-type have been implicated in infection in 18% of included pathogens, *bo_3_*-type in 14%, and *cbb_3_*-type in 9%, alone or in conjunction with *bd*-type COs.

**TABLE 2:** Bacterial pathogens in which COs have been found to be important for infection

Species	Total COs^*a*^	Relevant human infection site	Infection-related COs	Description of requirement
*Aggregatibacter actinomycetemcomitans*	1	Oral cavity	*bd*-type	Essential in both mono-infection and co-infection in a murine abscess model, as determined via transposon screen (31).
*Bordetella bronchiseptica*	7 (1)	Respiratory tract	*bo_3_-type*	*bo_3_*-type was sufficient for wild-type levels of persistence and colonization in murine respiratory infection models (32).
*Bordetella pertussis*	3 to 4	Respiratory tract	*bo_3_-*type *aa_3_*-type	Identified in transposon screen to find factors required during murine infection (33).
*Brucella abortus*	4^*b*^	Systemic	*bd-type*	Identified in transposon screens to find factors required for intracellular survival (34, 35). Loss of *bd-*type CO resulted in severe attenuation in intraperitoneally infected mice (34, 35).
*Burkholderia pseudomallei*	9^*b*^	Cystic fibrosis respiratory tract	*bd-*type	Strains recovered from chronically infected patients had increased expression of *bd-*type CO-encoding genes when grown in synthetic CF sputum media (36).
		Systemic	*bd-*type	Bacteria grown in human sera and recovered from infected mouse spleens had increased expression as compared to those grown in soil-mimicking media (37, 38).
*Escherichia coli*	3	Gastrointestinal tract	*bd-*type	In competition experiments, aerobic respiration broadly and *bd*-I more specifically were required to compete with wild-type bacteria for colonization (39).
		Urinary tract	*bd-*type	Cytochrome *bd*-I was highly expressed in strains isolated from patients with UPEC urinary tract infections (40). Loss of *bd*-I affected the ability of strains to compete with wild-type bacteria in a mouse UTI colonization model (41). *bd*-I was required for both intracellular and extracellular survival in the bladder (42). When the bacteria were extracellular, *bd*-I helped protect against nitric oxide and against innate immune cells (41). When the bacteria were intracellular, *bd*-I impaired the respiration efficiency of the host cell mitochondria, altered host cell metabolism, and decreased host cell apoptosis (42).
*Klebsiella pneumoniae*	3	Opportunistic; gastrointestinal tract	*bd-*type	Mice infected intraperitoneally with a strain lacking cytochrome *bd*-II had increased survival relative to mice infected with the wild-type strain, as well as lower bacterial burden in the blood, liver, spleen, and lung (43). While *bd-II* was not critical for intragastric infection in wild-type mice, loss of *bd-II* resulted in attenuation in an Inflammatory bowel disease (IBD) model (43).
*Listeria monocytogenes*	2^*b*^	Gastrointestinal tract	*bd-*type *aa_3_*-type	Aerobic respiration was required for wild-type levels of intracellular growth and murine intragastric infection (44). Both COs were required during murine infection, with loss of the *bd*-type CO resulting in more attenuation than loss of the *aa_3_*-type CO (45). The *bd*-type CO was required for intracellular growth in tissue culture cells (45, 46). Regenerating NAD^+^ during infection via alternative pathways partially rescues the infection defect seen with strains that are unable to respire (47).
*Mycobacterium marinum*	2 (2)	Skin; model for *M. tuberculosis* in fish	*bd-*type	Expression of *bd*-type CO was induced in mouse macrophages and in a zebrafish embryo infection model (48).
*Mycobacterium smegmatis*	3 (2)	Nonpathogenic; model for *M. tuberculosis*	*bd*-type	*bd*-type CO was important for growth in low oxygen (49). The loss of *bd*-type CO resulted in increased susceptibility to hydrogen peroxide (50). Overexpression of *bd*-type CO rescued the *in vitro* defect caused by loss of the *bc_1_/aa_3_*-type CO (51).
*Mycobacterium tuberculosis*	2	Respiratory tract	*bd-*type	The loss of the *bd*-type CO resulted in attenuation during competition with wild-type bacteria during murine aerosol infection (52). Attenuation was often, but not always, seen in mouse mono-infection (53–55). The *bd-*type CO was important for survival within the host later in infection after the initiation of the adaptive immune response and INF-gamma signaling (52, 53). This requirement corresponded with an increase in gene expression (53).
			*bc_1_/aa_3_*-type	Dispensable for growth in a mouse model (54). Expression of the genes encoding the *aa_3_-*type CO decreased across the course of infection (53). Inhibition in a non-human primate model halted disease progression but did not affect the bacteria already within granulomas (54).
*Mycobacterium ulcerans*	1	Skin and soft tissue	*bc_1_/aa_3_*-type	Chemical inhibition of the *bc_1_-aa_3_* complex resulted in clearance in a mouse footpad infection model (56).
*Pseudomonas aeruginosa*	5 (4)	Opportunistic pathogen	*cbb_3_*-type	Important for biofilm formation and virulence in *C. elegans (57*).
*Rickettsia conorii*	2	Systemic	*bd-type*	The genes encoding the *bd*-type CO are upregulated during murine infection, as measured in lung and brain tissues (58).
*Salmonella enterica* serovar Enteritidis	3	Gastrointestinal tract	*bd*-type	Identified as differentially expressed between high-pathogenicity and low-pathogenicity strains, having a significantly higher level of expression in highly virulent strains as opposed to less virulent strains (59).
*Salmonella enterica* serovar Typhimurium	3	Gastrointestinal tract	*bd*-type	Required for effective competition with wild-type bacteria during oral or systemic infection in mice, either via oral or intraperitoneal inoculation (60–62). Strains lacking *bd*-type COs were highly attenuated in a newly hatched chicken model and attenuated in an oral inoculation mouse model (63). *bd-*type COs contributed to defense against the nitric oxide produced by the immune system (61) and were important for survival within chicken macrophages (63). Cytochrome *bd-II* was important for post-antibiotic expansion in the intestines and was required for fecal-oral transmission in mice (62).
			*bo_3_-type*	Required for effective competition with wild-type bacteria during systemic infection in mice either via oral or intraperitoneal inoculation (60). Strains lacking the *bo_3_*-type CO were somewhat attenuated in a newly hatched chicken model (63, 64) and were negatively selected for during a single gene deletion screen in intraperitoneally infected mice (65).
*Shigella flexneri*	2	Gastrointestinal tract	*bd*-type	Cytochrome *bd-*II was required for intracellular survival and virulence in tissue culture cells and in mice inoculated intranasally (66), as well as intrarectally-infected guinea pigs (67). It was required to deplete the oxygen within the host mucosa, which was required for forming foci of infection and thus colonizing the tissue (67).
*Staphylococcus aureus*	2	Systemic	*bd*-type	Aerobic respiration was required for systemic infection in mice (68). The *bd*-type CO was required for colonizing the heart in this model.
			*aa_3_-type*	An *aa_3_*-type cytochrome oxidase was required for colonizing the liver in a mouse systemic infection model (68).
*Streptococcus agalactiae*	1	Systemic	*bd*-type	Loss of *bd*-type CO resulted in reduced growth in human blood and increased survival of neonatal rats in a sepsis model (69), as well as reduced colonization of the heart, kidney, and brain in a systemic mouse model (70).
*Vibrio cholerae*	4	Gastrointestinal tract	*bd*-type	Found in a transposon screen to find factors that are important for colonization in an infant rabbit model (71). Aerobic respiration was required for colonization of the small intestine in an infant mouse model (72, 73). Cytochrome *bd-*I was required for effective competition with wild-type bacteria in this model and was sufficient for WT-levels of colonization of the small intestine (73).
			*cbb_3_*-type	Found in a transposon screen to find factors that are important for colonization in an infant rabbit model (71). A strain with only *cbb_3_* was able to colonize the intestine in an infant mouse model but was defective relative to wild-type bacteria (73).
			*bd-*type & *cbb_3_*-type	While neither cytochrome oxidase was required for mono-infection, a strain lacking both *bd-I* and *cbb_3_* was unable to colonize the intestine in an infant mouse model (73).

^*a*^ Number of operons containing a full set of cytochrome oxidase-encoding genes. If lone subunits are found in the genome, the number of lone subunits is added in parentheses.

^*b*^ This number was determined by the authors using available genome annotations rather than previous publications.

Looking further into the characteristics of the 21 different bacterial pathogens studied (Table 3), additional trends emerge. The vast majority of investigated species (74%) are facultative anaerobes and therefore do not rely exclusively on aerobic respiration to survive. Out of the five obligate aerobes that have been studied, four are *Mycobacterium* spp. and therefore closely related. This limited sample makes drawing broader conclusions about the roles that COs play in obligate aerobe pathogens challenging. Additionally, the majority of studied pathogens infect tissues with reduced oxygen availability, especially the gastrointestinal tract (33% of pathogens investigated). In environments with varying oxygen availability, the purpose of the requirement of different COs, especially the high-affinity antimicrobial-resistant *bd*-type CO, is likely to vary.

**TABLE 3:** Infection characteristics of bacterial pathogens whose virulence depends on COs

Species	Gram stain	Obligate aerobe?	Relevant human infection site	Intracellular lifestyle during infection?	Infection-related COs
*Aggregatibacter actinomycetemcomitans*	Negative	No	Oral cavity	No	*bd*-type
*Bordetella bronchiseptica*	Negative	Yes	Respiratory tract	No	*bo_3_-type*
*Bordetella pertussis*	Negative	Yes	Respiratory tract	No	*bo_3_-*type *aa_3_*-type
*Brucella abortus*	Negative	No	Systemic	Yes	*bd-type*
*Burkholderia pseudomallei*	Negative	No	Cystic fibrosis respiratory tract	Yes	*bd-*type
			Systemic	Yes	*bd-*type
*Escherichia coli*	Negative	No	Gastrointestinal tract	No	*bd-*type
			Urinary tract	Yes	*bd-*type
*Klebsiella pneumoniae*	Negative	No	Opportunistic	Yes	*bd*-type
*Listeria monocytogenes*	Positive	No	Gastrointestinal tract	Yes	*bd-*type; *aa_3_*-type
*Mycobacterium marinum*	Acid-fast	Yes	Skin; model for *M. tuberculosis* in fish	Yes	*bd-*type
*Mycobacterium smegmatis*	Acid-fast	Yes	Nonpathogenic; model for *M. tuberculosis*	Yes	*bd*-type
*Mycobacterium tuberculosis*	Acid-fast	Yes	Respiratory tract	Yes	*bd-*type
*Mycobacterium ulcerans*	Acid-fast	Yes	Skin and soft tissue	Yes	*bc_1_-aa_3_*-type
*Pseudomonas aeruginosa*	Negative	No	Opportunistic pathogen	*No*	*cbb_3_*-type
*Rickettsia conorii*	Negative	No	Systemic	*Yes*	*bd-type*
*Salmonella enterica* serovar Enteritidis	Negative	No	Gastrointestinal tract	*Yes*	*bd*-type
*Salmonella enterica* serovar Typhimurium	Negative	No	Gastrointestinal tract	*Yes*	*bd*-type; *bo_3_-type*
*Shigella flexneri*	Negative	No	Gastrointestinal tract	*Yes*	*bd*-type
*Staphylococcus aureus*	Positive	No	Systemic	Yes	*bd*-type; *aa_3_-type*
*Streptococcus agalactiae*	Positive	No	Systemic	Yes	*bd*-type
*Vibrio cholerae*	Negative	No	Gastrointestinal tract	No	*bd*-type; *cbb_3_*-type

Another trend that emerges from Table 3 is the high percentage of pathogens that adopt an intracellular lifestyle during infection. While the majority of the bacteria are not obligate intracellular pathogens, most (76%) utilize an intracellular niche in a way critical to their pathogenicity. While living within a host cell offers advantages, including immune evasion, protection from antibiotics, and, in some cases, trafficking to new infection sites, intracellular bacteria face different environmental conditions than extracellular bacteria. These environments require distinct respiratory strategies, as shown for uropathogenic *E. coli* (discussed below in the section “Intracellular uropathogenic *E. coli* utilize a *bd*-type cytochrome oxidase not only to respire but also to manipulate the host cell metabolism to its advantage”).

## *BD-*TYPE CYTOCHROME OXIDASES HAVE UNIQUE PROPERTIES THAT ARE IMPLICATED IN THEIR ROLES IN VIRULENCE

As mentioned, *bd-*type COs are evolutionarily distinct from HCOs and have no sequence homology to HCOs (22). *bd-*type COs fall into at least three families and can be composed of two to four polypeptide chains (74). The core structure is comprised of two subunits, one of which coordinates the three hemes (two *b* hemes and one *d* heme) and contains the Q-loop, the quinol binding domain located within a hydrophilic loop. Unlike HCOs, *bd*-type COs do not pump protons across a membrane; instead, they generate a proton motive force through quinol oxidation, which releases protons outside the membrane, and oxygen reduction, which utilizes cytoplasmic protons (75–77).

While evidence suggests that HCOs contribute exclusively to energy production, *bd-*type COs fulfill other roles within the cell, including directly detoxifying some reactive oxygen and nitrogen species like hydrogen peroxide (78) and peroxynitrite (79). *bd-*type COs are also more resistant than HCOs to many antimicrobials encountered during infection, such as reactive oxygen and nitrogen species, which are effective inhibitors of HCOs (80). *bd*-type COs have also been found to be less sensitive than HCOs to hydrogen sulfide, which is found in high levels in the gut (81). In *E. coli*, the organism in which *bd*-type COs have been best characterized, expression of *cydAB*, the genes encoding the primary *bd*-type CO of this organism, is induced not only by low oxygen (82, 83) but also by other stressful conditions such as changes in pH (83, 84), high temperature (85), dissipation of the proton gradient (86), and the presence of toxins like potassium cyanide (87). Since CydAB is better able to handle these adverse conditions than the *bo_3_*-type CO primarily used under standard growth conditions, increased *cydAB* expression ensures that sufficient CydAB is present for *E. coli* to maintain respiration despite host attempts to kill the bacteria.

Since *bd-*type COs are important to virulence and unique to bacteria and archaea, these COs have been highlighted as potential drug targets (88, 89). Knowing that *bd*-type COs are important during infection does not, however, reveal the mechanisms behind their importance, which could affect the types of infections these drugs could effectively treat or the stage of infection when the drugs would be most impactful. When the mechanism for the *bd-*type COs’ role has been elucidated, the reason for the requirement has varied between organisms. For example, in *Listeria monocytogenes,* a *bd-*type CO was required to regenerate NAD^+^ from NADH (47). By contrast, in *Shigella flexneri*, aerobic respiration through *bd-*type COs was required to deplete oxygen and create the hypoxic conditions this pathogen needs for effective colonization of the colon mucosa (67). In most cases, however, the reason that a specific CO is required has not yet been determined. Research on two organisms, *M. tuberculosis* and uropathogenic *E. coli* (UPEC), highlights the developing mechanistic understanding of the roles *bd-*type COs play in infection and the promising potential of targeting them in treatment.

### Targeting a *bd*-type cytochrome oxidase in *Mycobacterium tuberculosis* is a promising direction for treating multidrug-resistant tuberculosis

*Mycobacterium tuberculosis,* the causative agent of tuberculosis, remains the leading cause of death by a single infectious agent globally, despite widespread efforts to combat this ancient disease (90). Indeed, the number of new cases annually has been rising since 2021 (90). While treatment options are available, the combination of unequal access to medical care, long treatment timelines, and increased incidence of multidrug-resistant bacteria has hindered progress toward eradicating tuberculosis (90).

A major factor contributing to the treatment failure of *M. tuberculosis* infection is the bacterium’s ability to enter a metabolically dormant, non-replicating state, which confers tolerance to many antibiotics (91–94). After initial infection, *M. tuberculosis* is also localized to granulomas, complex structures primarily composed of immune cells that encase the bacteria and infected cells, limiting access of antimycobacterial therapies to the bacteria (95). Current strategies employ long-term treatment to address these challenges, with the recommended duration being up to 6 months for antibiotic-susceptible tuberculosis and up to 2 years for multidrug-resistant tuberculosis (96). The treatments for multidrug-resistant *M. tuberculosis* are also associated with toxicity and have lower cure rates (96–98). Better treatments are required to end the tuberculosis epidemic.

In 2012, the Food and Drug Administration approved a new anti-tuberculosis drug, bedaquiline, that targets ATP synthase (99, 100). Excitingly, this drug can target and kill persister cells (17, 101–103) and can be used in combination with existing first-line antimicrobials used to treat tuberculosis (90). This discovery has opened a new avenue for drug development and has sparked interest in targeting the electron transport chain. The current state of research on *M. tuberculosis* bioenergetic inhibitors is reviewed in previous studies (104, 105).

**Fig 2:**
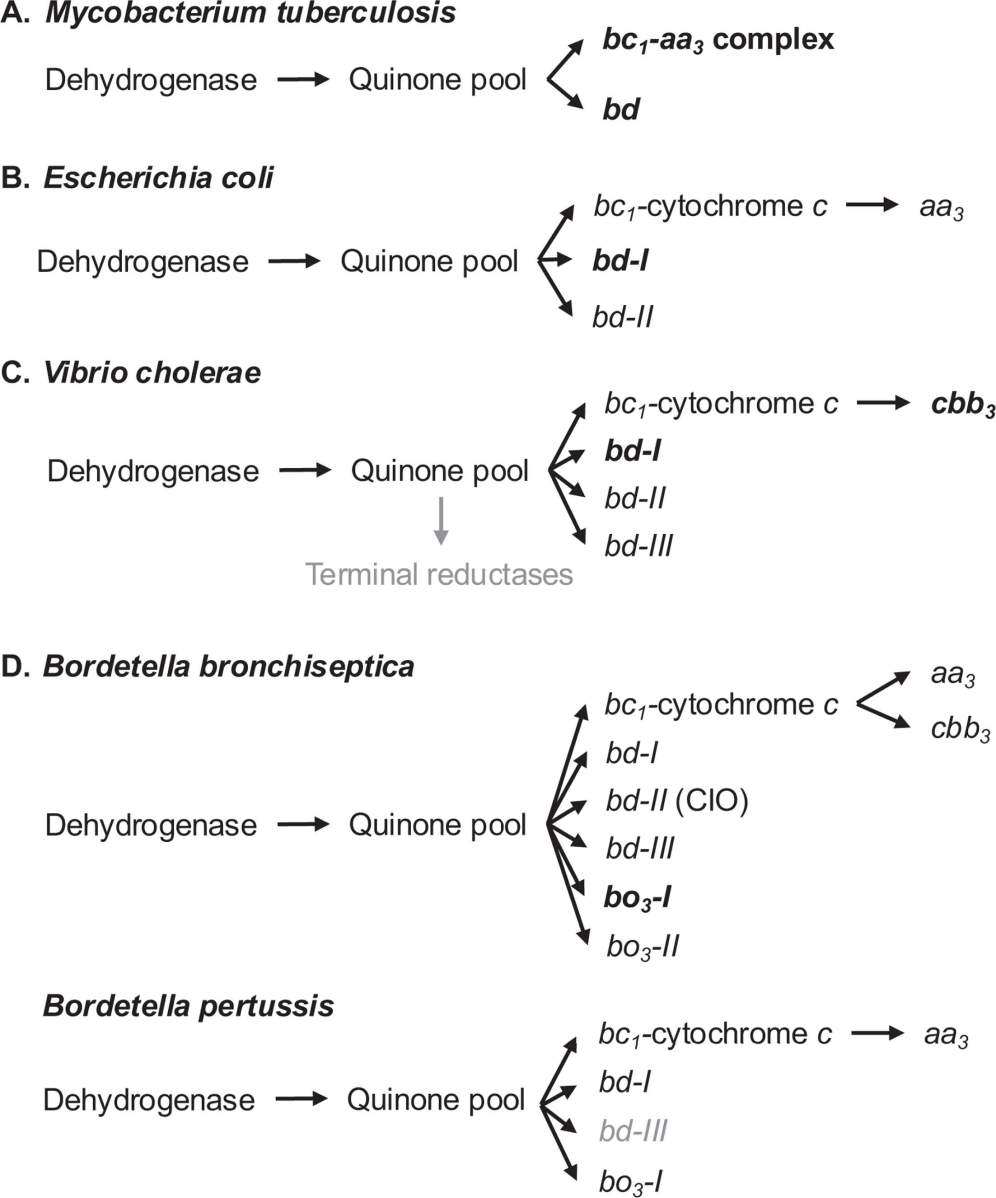
The simplified branched aerobic respiratory pathways of *M. tuberculosis* (**A**), *E. coli* (**B**), *Vibrio cholerae* (**C**), and *Bordetella bronchiseptica*/*Bordetella pertussis* (**D**). The cytochrome oxidases that are linked to virulence are shown in bold.

As an obligate aerobe, *M. tuberculosis* encodes a full suite of proteins involved in respiration, including two COs: an *aa_3_-*type CO (encoded by *ctaCDE*), which forms a supercomplex with cytochrome *bc_1_* (encoded by *qcrCAB*), and a *bd-*type CO (encoded by *cydAB*) (106) (Fig. 2A). *Mycobacterium marinum* and *Mycobacterium smegmatis,* two closely related species used as models for *M. tuberculosis,* encode these two COs and additional subunits. In all three species, the *bc_1_-aa_3_* supercomplex is the dominant CO under standard culturing conditions, while the *bd-*type CO is required for growth under low-oxygen conditions (49, 51, 107).

In animal models of tuberculosis, the *bd-*type CO seems to be more important than the *bc_1_-aa_3_* supercomplex. Expression of the *aa_3_-*type CO decreases over the course of infection within the mouse lung (53), and the *bc_1_-aa_3_* complex is not required for infection (54). Expression of the *bd-*type CO is induced during *M. marinum* infection of both mouse macrophages and zebrafish embryos (48) and during *M. tuberculosis* mouse infection (53). Additionally, mice infected with strains lacking the *bd-*type CO have lower bacterial burdens than mice infected with wild-type bacteria in most, but not all, studies (53–55). The virulence defect becomes more evident when the strain lacking the *bd-*type CO is competed against wild-type bacteria, an experimental design that ensures the two strains are exposed to identical conditions, including immune pressures and nutrient availability. In a competitive infection, the strain lacking the *bd-*type CO competed effectively with wild-type bacteria early in infection but was significantly defective later once the adaptive immune response was active (52). This defect depended on interferon signaling, which induces acidification of *M. tuberculosis*-containing phagosomes. Since the *aa_3_-*type CO is preferentially inhibited by acidic conditions (28, 78), strains lacking the *bd-*type CO were preferentially killed in the acidified phagosomes. Therefore, *M. tuberculosis* relies on a *bd-*type CO during infection because of its resistance to the antimicrobial actions of the adaptive immune response.

As with bedaquiline, the effects of drugs that target COs are also additive with other antimicrobials. Synthetic compounds have been developed to inhibit the *bc_1_-aa_3_* complex, including Q203, an anti-tuberculosis drug currently in clinical trials (108–111). Q203 cannot clear *M. tuberculosis* infection alone, as overexpression of the genes encoding the *bd*-type CO can compensate for loss of the *bc_1_-aa_3_* complex (51, 110). However, the combination of *bc_1_-aa_3_* complex inhibition and loss of the *bd*-type CO is synthetically lethal (52, 55, 107, 108). Loss of the *bd*-type CO or inhibition of the *bc_1_-aa_3_* complex is also synergistic with bedaquiline treatment (50, 103, 112) and could be combined with the current recommended anti-tuberculosis antibiotic regimen. Q203 has also been shown to be effective against *Mycobacterium ulcerans,* which causes severe skin and soft tissue infections that typically require extended antibiotic treatment to eradicate, as many strains of *M. ulcerans* only encode the *bc_1_-aa_3_* complex (56, 113).

Together, these efforts to combat the enduring threat of tuberculosis emphasize the power of targeting the electron transport chain broadly and COs more specifically to improve clinical outcomes by clearing persistent bacterial populations while also elucidating one mechanism for the role of *bd*-type COs in infection.

### Intracellular uropathogenic *E. coli* utilize a *bd*-type cytochrome oxidase not only to respire but also to manipulate the host cell metabolism to its advantage

Urinary tract infections (UTIs) are among the most common bacterial infections, annually affecting approximately 400 million people globally and costing approximately $340–$450 million per year in the US alone (114–117). UTIs are also one of the most common forms of nosocomial infections and are a common complication of hospitalization, especially following catheterization (118). The majority of UTIs are caused by uropathogenic *E. coli* (UPEC) (119, 120). As with many pathogens, increasing antibiotic resistance is a problem for treating UPEC infections (121).

During infection, UPEC is found both extracellularly, attached to the luminal epithelium in biofilms, and intracellularly within urothelial cells it invades. Once internalized in urothelial cells, the bacteria escape into the host cell cytoplasm and form multicellular biofilm-like intracellular bacterial communities (IBCs). In both intracellular and extracellular environments, UPEC is subject to low oxygen tension. Within the bladder, oxygen levels are between 4% and 7%, as compared to approximately 21% in ambient air (122). Intracellular UPEC reside within the hypoxic cytosol; while the exact oxygen levels in this environment are difficult to discern, UPEC upregulates factors important for resisting oxidative stress, indicating low oxygen availability (123). Although UPEC is a facultative anaerobe and utilizes alternative electron acceptors during infection, aerobic respiration is required for infection. Disruption of ubiquinone synthesis results in attenuation in both chronic and acute infection models, affecting both intracellular and extracellular bacterial burdens (124). The amount of biofilm formation, which protects the bacteria from external threats, also depends on oxygen availability, with the highest levels occurring in ambient air or at oxygen levels similar to those encountered in the bladder (125).

*E. coli* encodes the genes for three COs: one *bo_3_*-type CO, which serves as the primary CO under ambient air conditions, and two *bd-*type COs, encoded by *cydABX* and *appBC* (82, 126) (Fig. 2B). While aerobic respiration broadly is required for UPEC infection and the genes encoding *bd-*type and *bo_3_*-type COs are all highly expressed in samples taken from individuals with UTIs, the *bd*-type CO encoded by *cydABX* plays a greater role and is required by both extracellular and intracellular UPEC for both the establishment of infection and for persistent bacteriuria (40–42, 124, 127). Loss of this *bd-type* CO also negatively affects biofilm formation and morphology, including that of IBCs (42, 127).

In a recent study, researchers from Vanderbilt University investigated the mechanistic reasons behind the requirement of *bd*-type COs during infection (42). They introduced a mutation, K252A, into CydA that disrupts quinol binding and the interaction with heme *b*, therefore generating a *bd*-type CO that could still detoxify toxins like nitric oxide but lacked respiratory activity (128, 129). In mouse infections, a strain lacking the native *bd-*type CO but producing CydA K252A reached wild-type levels of bacterial burden when surviving extracellularly, but reached a lower bacterial burden relative to wild-type when surviving intracellularly. This suggests that in the extracellular environment, UPEC requires the *bd*-type CO to combat toxins such as nitric oxide, a role previously attributed to CydAB in UPEC (41), whereas within the intracellular environment, UPEC requires cytochrome *bd-*mediated oxidation.

The researchers then examined the effect of UPEC on the host urothelial cells. UPEC infection impaired mitochondrial efficiency in a cytochrome *bd-*dependent manner and altered mitochondrial organization by disrupting mitochondrial fusion, a process that normally occurs during intracellular infection (130). The authors hypothesized that the *bd-*type CO, which has approximately a 1,000-fold higher affinity for oxygen than human cytochrome *c* oxidase, reduced cytosolic oxygen availability to levels that triggered the host cell’s hypoxia response (131, 132). This hypothesis is supported by the increased levels of the hypoxia-inducible transcription factor HIF-1 in infected cells as compared to uninfected cells, which shifts the cell’s metabolism away from oxidative phosphorylation and toward aerobic glycolysis (123, 130). HIF-1 activation also antagonizes pro-apoptotic pathways (133), making non-intracellularly infected cells five times more likely to undergo apoptosis than those infected with UPEC.

In summary, we now know not only that the *bd-*type CO encoded by *cydAB* is required by UPEC during infection but also why. By utilizing a high-affinity CO, intracellular UPEC can use oxygen more efficiently than the host cell, which relies on a low-affinity CO. UPEC respiration therefore generates a highly hypoxic intracellular environment, triggering a signaling cascade that shifts the host cell metabolism and prevents host cell apoptosis. Given that a disproportionate number of intracellular pathogens require *bd*-type COs for infection, further work should investigate the generality of this finding.

## THE MECHANISTIC ROLE OF OTHER CYTOCHROME OXIDASES DURING INFECTION IS LESS CLEAR

Prioritizing research on *bd-*type COs is rational, given the available experimental evidence. The known unique characteristics of *bd-*type COs also guide inquiries into the mechanistic role these COs play during infection. Additionally, when considering antimicrobial development, the risk of off-target effects is greater when targeting HCOs, which encompass the cytochrome oxidases used by eukaryotes in their mitochondria, than when targeting *bd*-type COs, which are structurally distinct. In cases when other COs are required during infection, the reason for that requirement is less clear. Beyond differences in oxygen affinity, electron donors, hemes utilized, and gene expression and protein production, it is not clear why one HCO would be more important than another during infection. Since bacterial respiratory chains often exhibit some redundancy even during infection, understanding the mechanistic roles of HCOs during infection, even when they are not the primary CO required, is essential for understanding bacterial aerobic respiration during infection.

### Both *bd*-type and *cbb*_3_-type cytochrome oxidases are important for *V. cholerae* colonization of the small intestine

The diarrheal disease cholera, caused by the gram-negative facultative anaerobe *V. cholerae,* remains a public health threat despite centuries of effort to combat it. In 2023, over half a million cholera cases were reported to the World Health Organization (134). The number of reported cases has been rising in recent years, and many factors, including insufficient global investment into safe water, sanitation, and hygiene (WASH) measures, climate change increasing *V. cholerae* range and seasonal abundance, and population migration and displacement, have indicated that we are unlikely to reach the goal of eliminating cholera as a public health threat in most countries by 2030 (134–136).

Like tuberculosis, cholera is a persistent problem in many resource-limited regions, where poverty and insufficient access to healthcare and public health measures make controlling outbreaks challenging. Additionally, *V. cholerae* freshly shed by infected individuals is hypervirulent, placing medical personnel and cohabitants at increased risk of infection once an outbreak begins (137–140). While oral cholera vaccines have been developed and are effective at controlling outbreaks when used in combination with WASH measures, the supply is insufficient to meet demand, and the protection they provide is short-lived (141). Once a patient is infected, treatment options are limited and focused on rehydration, with antibiotics recommended only for patients with severe dehydration (142–144).

The majority of *V. cholerae* strains are nonpathogenic and live within aquatic environments (145). The subset that causes cholera belongs to two serogroups: O139, which emerged in 1992 and is now functionally extinct (146–148), and O1, which can be further subdivided into classical strains, responsible for the first six pandemics of cholera, and El Tor strains, responsible for the ongoing seventh pandemic (149–151). Within a human host, *V. cholerae* colonizes the gastrointestinal tract, which is broadly considered physiologically hypoxic. However, the intestines have steep oxygen gradients, both longitudinally along the length of the organ and radially between the lumen and epithelial cell surface (152). *V. cholerae* localizes to the epithelium, giving the bacteria more access to oxygen than they would have within the lumen (153, 154). Infection can also impact the levels of available oxygen. In the gastrointestinal tract, increased inflammation has been associated with increased bacterial respiration, favoring facultative aerobes (155).

*V. cholerae* has four COs for aerobic respiration: one *cbb_3_-*type and three *bd*-type, labeled *bd-I, bd-II,* and *bd-III* (156) (Fig. 2C). *V. cholerae* also encodes four terminal reductases for anaerobic respiration that allow this species to use nitrate, fumarate, trimethylamine-N-oxide, and biotin sulfoxide as alternative terminal electron acceptors to oxygen (157–160). During infection, both aerobic and anaerobic metabolism contribute to the high bacterial burdens achieved, although aerobic metabolism has been shown to be the primary driver in the infant mouse model of infection (72).

Van Alst, Demey, and DiRita (73) investigated the roles of COs and terminal reductases in *V. cholerae* during infection. They found that aerobic respiration is required for colonization of the small intestine in an infant mouse model, while anaerobic respiration is not. No individual CO was required during infection with a single strain. However, by using Comparative Multiplex PCR Amplicon Sequencing (CoMPAS), which combines transposon sequencing (Tn-seq), multiplex genome editing by natural transformation, and competitive infections in mice, they were able to determine that *bd-I* is important during infection, as losing *bd-I* resulted in a competitive disadvantage relative to wild-type bacteria.

Based on *in vitro* expression data, they predicted that *bd-I* and *cbb_3_* would be important during infection, despite the fact that deleting *cbb_3_* did not confer a competitive disadvantage relative to wild-type bacteria in the CoMPAS analysis. This prediction was reinforced by a previous Tn-seq experiment in an infant rabbit model that identified factors required for colonization, which found that loss of *cbb_3_* and *bd-I*, as well as *bd-III*, reduced the bacteria’s ability to colonize (71). To test this hypothesis, Van Alst et al. infected mice with a strain lacking both *cbb_3_* and *bd-I,* or with strains that had only *cbb_3_* or only *bd-I*. Mice infected with a strain with only *bd-I* reached wild-type levels of bacterial burdens in the intestine, indicating that *bd-I* is sufficient for colonization. A strain with only *cbb_3_* was able to colonize the intestine but was still defective relative to wild-type bacteria, especially when mice were inoculated with anaerobically grown *V. cholerae* cultures rather than aerobically grown cultures. Finally, the strain lacking both *cbb_3_* and *bd-I* was unable to colonize the mice. These results indicate that *bd-I* is the primary CO required during infection but that both *bd-*type and *cbb_3_*-type COs are required for *V. cholerae* to colonize the small intestine. Therefore, efforts to combat *V. cholerae* infection would require targeting not just *bd-I* but also *cbb_3_* to be effective. Further research will be required to untangle the individual contributions of *bd-I* and *cbb_3_*.

### A *bo*_3_-type cytochrome oxidase is the most important cytochrome oxidase for *Bordetella* infection of the mammalian respiratory tract

Despite widespread vaccination, pertussis, or whooping cough, remains an ongoing public health problem. Characterized by a violent, rapid, repeated cough followed by a gasping inhale, resulting in the eponymous “whooping” sound, pertussis can take weeks to months to resolve and can lead to complications, including broken ribs and pneumonia (161). Before the introduction of whole-cell pertussis vaccines in the US in the 1940s, pertussis was a common childhood illness and a major contributor to childhood mortality. The switch from whole-cell to acellular pertussis vaccines in many countries, including the United States, due to safety concerns with whole-cell vaccines, has been associated with increased reported pertussis incidence (162–165). Infants under 1 year of age have the highest reported rate of pertussis and are also at the highest risk of severe disease and death due to their immature immune systems (165). However, due to the waning immunity conferred by current acellular vaccines, an increasing number of adolescents and young adults are also being affected by pertussis (166–168). Although acellular pertussis vaccines use proteins critical for virulence as antigens, immunization fails to elicit the immune response required for long-term protection against both disease and colonization (169, 170). Given the limitations of the acellular vaccine and the adverse side effects associated with the whole-cell vaccine, new treatments are needed to combat the return of pertussis.

The classical Bordetellae, comprising *B. pertussis, B. parapertussis*_Hu_*,* and *B. bronchiseptica*, are gram-negative respiratory pathogens. *B. pertussis*, the causative agent of pertussis, and *B. parapertussis*_Hu_, which causes pertussis-like disease, are human-specific pathogens, while *B. bronchiseptica* has a wide host range; it can infect almost all mammals, including mice, and has also been isolated from the environment (171). Best known for causing kennel cough in dogs, *B. bronchiseptica* predominantly causes chronic and often asymptomatic infections, while *B. pertussis* causes acute disease. Despite their different hosts and infection characteristics, *B. bronchiseptica* and *B. pertussis* encode nearly identical virulence factors, many of which are functionally interchangeable (172–176). Genomic analyses indicate that *B. pertussis* evolved from a *B. bronchiseptica*-like ancestor and that specializing for a human host coincided with significant genome reduction (176). B. *bronchiseptica,* therefore, can be used as a powerful model for understanding both *B. pertussis* pathogenesis and evolution, allowing us to use a native infection model to understand the role of different factors during *Bordetella* infection.

*B. bronchiseptica* is predicted to encode seven complete COs and one orphan CO catalytic subunit. These putative COs have not been biochemically characterized; instead, they have been computationally annotated based on the similarity of the genes that encode them to characterized CO-encoding genes. Based on these predictions, *B. bronchiseptica* encodes one *cbb_3_*-type CO (encoded by *ccoNOQP*)*,* two class *bd-*type COs (encoded by *cydAB1* and *cydAB3*), one CIO-type CO (encoded by *cioAB*), two *bo_3_-*type COs (encoded by *cyoABCD1* and *cyoABCD2*), one *aa_3_-*type CO (encoded by *ctaCDFGE1*), and one lone *aa_3_-*type catalytic subunit (encoded by *ctaD2*) (Fig. 2D). By contrast, *B. pertussis* is predicted to encode either three or four of the eight COs encoded by *B. bronchiseptica*, depending on the strain: one or two *bd-*type (CydAB1 and sometimes CydAB3), one *bo_3_*-type (CyoABCD1), and one *aa_3_*-type (CtaCDFGE1).

In a recent publication (32), we investigated the role that these different COs play during infection. While the respiratory tract has greater oxygen availability than other host locations, such as the digestive tract, it still encompasses a variety of microenvironments whose conditions are difficult to assess. Since *B. bronchiseptica* adheres to the ciliated epithelial cells lining the respiratory tract (177), these bacteria are most likely not exposed to the air their host breathes in and out. Instead, they lie beneath the layer of mucus and tethered mucins secreted by the ciliated epithelial cells and neighboring goblet cells to protect the host from airborne particulates and microbes. Given that oxygen must diffuse through the mucus layer and that bacteria compete with host cells for that oxygen, we hypothesized that *B. bronchiseptica* may require high-affinity COs in this environment to maintain respiration. Given the evidence in other organisms, we suspected that a *bd*-type CO would be most important. However, work in *B. pertussis,* using Tn-seq to identify genes that are conditionally essential during murine infection, indicated that both the *bo_3_*-type and *aa_3_*-type COs are needed in the respiratory tract environment (33).

Using *B. bronchiseptica* strains with deletions of one or more cytochrome oxidase-encoding gene loci, we investigated the roles of the different COs during murine infection. No individual CO was required for infection. We then focused on the COs encoded in *B. pertussis,* hypothesizing that since *B. pertussis* is found exclusively within the human respiratory tract, this species has maintained only the COs required for survival within a host and no longer faced selective pressure to maintain those required for survival in other environments. We were able to make three strains that only encoded a single CO: *cyd1^+^,* which only encoded a *bd*-type CO; *cta1^+^,* which only encoded an *aa_3_*-type CO; and *cyo1^+^,* which only encoded a *bo_3_*-type CO. Surprisingly, mice infected with *cyo1^+^* exhibited the same bacterial burden as mice infected with wild-type bacteria in two different models of mouse infection: one designed to test the ability to establish infection and the other to test persistence once introduced throughout the respiratory tract. These data indicate that the *bo_3_-*type CO, despite being low-affinity, is sufficient for murine infection. Both *cyd1^+^* and *cta1^+^* were defective relative to wild-type bacteria in both infection models; however, both were able to infect and maintain detectable bacterial burden in the mice through the final time point assessed. Therefore, a *bo_3_-*type CO is the primary CO used by *B. bronchiseptica* during murine infection, while *bd*-type and *aa_3_-*type COs appear to fill auxiliary roles.

Unlike most other organisms studied, a *bd*-type CO is not the primary CO required for *B. bronchiseptica* infection. Therefore, targeting *bd*-type COs with drugs is unlikely to be effective in this organism. Given the lack of phenotypes observed when deleting the genes encoding individual COs, clearing *B. bronchiseptica* infections would likely require targeting all three COs conserved in *B. pertussis*.

## THERAPEUTIC POTENTIAL AND CHALLENGES OF INHIBITING CYTOCHROME OXIDASES

Research on *M. tuberculosis* has shown that targeting the ETC broadly and COs specifically can address ongoing challenges posed by treatment-resistant bacterial populations. These findings revitalized interest in investigating COs and their roles during infection, revealing that *bd-*type COs are most commonly required (Table 1). Targeting *bd-*type COs, however, is unlikely to work on its own. The flexibility of bacterial respiratory chains means that most bacteria encode the potential to make multiple COs, enabling survival even when the CO preferentially used in a given environment is inhibited. Indeed, this flexibility is evident in most bacteria whose COs have been investigated. Generally, the loss of a single CO does not significantly affect the course of infection in mono-infection and does not result in full attenuation even in competition experiments. In both *M. tuberculosis* and *V. cholerae,* targeting the *bd*-type CO alone was not enough to prevent infection (53–55, 73). Instead, drugs targeting COs will likely be most effective when used in combination with conventional antimicrobials to enhance their efficacy.

Currently, our understanding of the role of COs during infection remains limited to a small selection of bacterial pathogens. Further research is required to determine whether the trends emerging from current studies are broadly applicable. For example, are *bd*-type COs of intracellular pathogens able to reprogram the metabolism of host cells in general, or is this a unique trait of the *bd-*type CO of UPEC (42)? Are obligate aerobic bacteria more likely to rely on HCOs than facultative anaerobes, or is this an unusual trait of the classical Bordetellae (32)? By understanding not only which COs are important for each pathogen but also why, we should be able to combine treatments that leverage bacterial physiology to our advantage. Priority should be given to pathogens that pose the greatest threat, particularly the ESKAPE pathogens and species and genera designated as Urgent Threats by the Centers for Disease Control and Prevention, which most require new strategies to combat these multidrug-resistant infections (178, 179). By following the example of *M. tuberculosis*, we may not only better understand bacterial physiology during infection but also find new ways to treat devastating diseases.

## References

[1] Mates SM, Eisenberg ES, Mandel LJ, Patel L, Kaback HR, Miller MH. 1982. Membrane potential and gentamicin uptake in Staphylococcus aureus. Proc Natl Acad Sci USA 79:6693–6697. doi:10.1073/pnas.79.21.6693 6959147 PMC347195

[2] Gunnison JB, Fraher MA, Jawetz E. 1964. Persistence of Staphylococcus aureus in penicillin in vitro. J Gen Microbiol 35:335–349. doi:10.1099/00221287-35-2-335 14179679

[3] Mayhall CG, Medoff G, Marr JJ. 1976. Variation in the susceptibility of strains of Staphylococcus aureus to oxacillin, cephalothin, and gentamicin. Antimicrob Agents Chemother 10:707–712. doi:10.1128/AAC.10.4.707 984804 PMC429818

[4] Newsom SWB. 1970. Staphylococcal persisters grown from empyema fluid on L-form medium. J Med Microbiol 3:669–673. doi:10.1099/00222615-3-4-669 5505023

[5] Lechner S, Lewis K, Bertram R. 2012. Staphylococcus aureus persisters tolerant to bactericidal antibiotics. J Mol Microbiol Biotechnol 22:235–244. doi:10.1159/000342449 22986269 PMC3518770

[6] Johnson PJT, Levin BR. 2013. Pharmacodynamics, population dynamics, and the evolution of persistence in Staphylococcus aureus. PLoS Genet 9:e1003123. doi:10.1371/journal.pgen.1003123 23300474 PMC3536638

[7] Brooun A, Liu S, Lewis K. 2000. A dose-response study of antibiotic resistance in Pseudomonas aeruginosa biofilms. Antimicrob Agents Chemother 44:640–646. doi:10.1128/AAC.44.3.640-646.2000 10681331 PMC89739

[8] Spoering AL, Lewis K. 2001. Biofilms and planktonic cells of Pseudomonas aeruginosa have similar resistance to killing by antimicrobials. J Bacteriol 183:6746–6751. doi:10.1128/JB.183.23.6746-6751.2001 11698361 PMC95513

[9] Harrison JJ, Turner RJ, Ceri H. 2005. Persister cells, the biofilm matrix and tolerance to metal cations in biofilm and planktonic Pseudomonas aeruginosa. Environ Microbiol 7:981–994. doi:10.1111/j.1462-2920.2005.00777.x 15946294

[10] Möker N, Dean CR, Tao J. 2010. Pseudomonas aeruginosa increases formation of multidrug-tolerant persister cells in response to quorum-sensing signaling molecules. J Bacteriol 192:1946–1955. doi:10.1128/JB.01231-09 20097861 PMC2838031

[11] Nguyen D, Joshi-Datar A, Lepine F, Bauerle E, Olakanmi O, Beer K, McKay G, Siehnel R, Schafhauser J, Wang Y, et al.. 2011. Active starvation responses mediate antibiotic tolerance in biofilms and nutrient-limited bacteria. Science 334:982–986. doi:10.1126/science.1211037 22096200 PMC4046891

[12] Keren I, Minami S, Rubin E, Lewis K. 2011. Characterization and transcriptome analysis of Mycobacterium tuberculosis persisters. mBio 2:e00100-11. doi:10.1128/mBio.00100-11 21673191 PMC3119538

[13] Parbhoo T, Mouton JM, Sampson SL. 2022. Phenotypic adaptation of Mycobacterium tuberculosis to host-associated stressors that induce persister formation. Front Cell Infect Microbiol 12:956607. doi:10.3389/fcimb.2022.956607 36237425 PMC9551238

[14] Keren I, Kaldalu N, Spoering A, Wang Y, Lewis K. 2004. Persister cells and tolerance to antimicrobials. FEMS Microbiol Lett 230:13–18. doi:10.1016/S0378-1097(03)00856-5 14734160

[15] Keren I, Shah D, Spoering A, Kaldalu N, Lewis K. 2004. Specialized persister cells and the mechanism of multidrug tolerance in Escherichia coli. J Bacteriol 186:8172–8180. doi:10.1128/JB.186.24.8172-8180.2004 15576765 PMC532439

[16] Shah D, Zhang Z, Khodursky AB, Kaldalu N, Kurg K, Lewis K. 2006. Persisters: a distinct physiological state of E. coli. BMC Microbiol 6:53. doi:10.1186/1471-2180-6-53 16768798 PMC1557402

[17] Koul A, Vranckx L, Dendouga N, Balemans W, Van den Wyngaert I, Vergauwen K, Göhlmann HWH, Willebrords R, Poncelet A, Guillemont J, et al.. 2008. Diarylquinolines are bactericidal for dormant mycobacteria as a result of disturbed ATP homeostasis. J Biol Chem 283:25273–25280. doi:10.1074/jbc.M803899200 18625705

[18] Berry S. 2003. Endosymbiosis and the design of eukaryotic electron transport. Biochim Biophys Acta 1606:57–72. doi:10.1016/s0005-2728(03)00084-7 14507427

[19] Calhoun MW, Thomas JW, Gennis RB. 1994. The cytochrome oxidase superfamily of redox-driven proton pumps. Trends Biochem Sci 19:325–330. doi:10.1016/0968-0004(94)90071-x 7940677

[20] Sousa FL, Alves RJ, Ribeiro MA, Pereira-Leal JB, Teixeira M, Pereira MM. 2012. The superfamily of heme–copper oxygen reductases: types and evolutionary considerations. Biochim Biophys Acta 1817:629–637. doi:10.1016/j.bbabio.2011.09.020 22001780

[21] Hemp J, Robinson DE, Ganesan KB, Martinez TJ, Kelleher NL, Gennis RB. 2006. Evolutionary migration of a post-translationally modified active-site residue in the proton-pumping heme-copper oxygen reductases. Biochemistry 45:15405–15410. doi:10.1021/bi062026u 17176062 PMC2535580

[22] Borisov VB, Gennis RB, Hemp J, Verkhovsky MI. 2011. The cytochrome bd respiratory oxygen reductases. Biochim Biophys Acta 1807:1398–1413. doi:10.1016/j.bbabio.2011.06.016 21756872 PMC3171616

[23] Preisig O, Zufferey R, Thöny-Meyer L, Appleby CA, Hennecke H. 1996. A high-affinity cbb_3_-type cytochrome oxidase terminates the symbiosis-specific respiratory chain of Bradyrhizobium japonicum. J Bacteriol 178:1532–1538. doi:10.1128/jb.178.6.1532-1538.1996 8626278 PMC177835

[24] D’mello R, Hill S, Poole RK. 1996. The cytochrome bd quinol oxidase in Escherichia coli has an extremely high oxygen affinity and two oxygen-binding haems: implications for regulation of activity in vivo by oxygen inhibition. Microbiology (Reading) 142:755–763. doi:10.1099/00221287-142-4-755 8936304

[25] Morris RL, Schmidt TM. 2013. Shallow breathing: bacterial life at low O_2_. Nat Rev Microbiol 11:205–212. doi:10.1038/nrmicro2970 23411864 PMC3969821

[26] Han H, Hemp J, Pace LA, Ouyang K, Ganesan K, Daldal JH, Daldal F, Blanke SR, Gennis RB. 2011. Adaptation of aerobic respiration to low O_2_ environments. Proc Natl Acad Sci USA 108:14109–14114. doi:10.1073/pnas.1018958108 21844375 PMC3161551

[27] Cunningham L, Pitt M, Williams HD. 1997. The cioAB genes from Pseudomonas aeruginosa code for a novel cyanide-insensitive terminal oxidase related to the cytochrome bd quinol oxidases. Mol Microbiol 24:579–591. doi:10.1046/j.1365-2958.1997.3561728.x 9179851

[28] Cooper M, Tavankar GR, Williams HD. 2003. Regulation of expression of the cyanide-insensitive terminal oxidase in Pseudomonas aeruginosa. Microbiology (Reading, Engl) 149:1275–1284. doi:10.1099/mic.0.26017-0 12724389

[29] Jackson RJ, Elvers KT, Lee LJ, Gidley MD, Wainwright LM, Lightfoot J, Park SF, Poole RK. 2007. Oxygen reactivity of both respiratory oxidases in Campylobacter jejuni: the cydAB genes encode a cyanide-resistant, low-affinity oxidase that is not of the cytochrome bd type. J Bacteriol 189:1604–1615. doi:10.1128/JB.00897-06 17172349 PMC1855770

[30] Miura H, Mogi T, Ano Y, Migita CT, Matsutani M, Yakushi T, Kita K, Matsushita K. 2013. Cyanide-insensitive quinol oxidase (CIO) from Gluconobacter oxydans is a unique terminal oxidase subfamily of cytochrome bd. J Biochem 153:535–545. doi:10.1093/jb/mvt019 23526305

[31] Lewin GR, Stacy A, Michie KL, Lamont RJ, Whiteley M. 2019. Large-scale identification of pathogen essential genes during coinfection with sympatric and allopatric microbes. Proc Natl Acad Sci USA 116:19685–19694. doi:10.1073/pnas.1907619116 31427504 PMC6765283

[32] McKay LS, Spandrio AR, Johnson RM, Sobran MA, Marlatt SA, Mote KB, Dedloff MR, Nash ZM, Julio SM, Cotter PA. 2024. Cytochrome oxidase requirements in Bordetella reveal insights into evolution towards life in the mammalian respiratory tract. PLoS Pathog 20:e1012084. doi:10.1371/journal.ppat.1012084 38976749 PMC11257404

[33] Gonyar LA, Gelbach PE, McDuffie DG, Koeppel AF, Chen Q, Lee G, Temple LM, Stibitz S, Hewlett EL, Papin JA, et al.. 2019. In vivo gene essentiality and metabolism in Bordetella pertussis. mSphere 4:e00694-18. doi:10.1128/mSphere.00694-18 31118307 PMC6531889

[34] Endley S, McMurray D, Ficht TA. 2001. Interruption of the cydB locus in Brucella abortus attenuates intracellular survival and virulence in the mouse model of infection. J Bacteriol 183:2454–2462. doi:10.1128/JB.183.8.2454-2462.2001 11274104 PMC95161

[35] Truong QL, Cho Y, Barate AK, Kim S, Hahn T-W. 2014. Characterization and protective property of Brucella abortus cydC and looP mutants. Clin Vaccine Immunol 21:1573–1580. doi:10.1128/CVI.00164-14 25253663 PMC4248770

[36] Price EP, Viberg LT, Kidd TJ, Bell SC, Currie BJ, Sarovich DS. 2018. Transcriptomic analysis of longitudinal Burkholderia pseudomallei infecting the cystic fibrosis lung. Microb Genom 4:e000194. doi:10.1099/mgen.0.000194 29989529 PMC6159556

[37] Kong C, Wong R-R, Ghazali A-K, Hara Y, Tengku Aziz TN, Nathan S. 2023. Transcriptional landscape of Burkholderia pseudomallei cultured under environmental and clinical conditions. Microb Genom 9:000982. doi:10.1099/mgen.0.000982 PMC10210952 37018040

[38] Ghazali A-K, Firdaus-Raih M, Uthaya Kumar A, Lee W-K, Hoh C-C, Nathan S. 2023. Transitioning from soil to host: comparative transcriptome analysis reveals the Burkholderia pseudomallei response to different niches. Microbiol Spectr 11:e0383522. doi:10.1128/spectrum.03835-22 36856434 PMC10100664

[39] Jones SA, Chowdhury FZ, Fabich AJ, Anderson A, Schreiner DM, House AL, Autieri SM, Leatham MP, Lins JJ, Jorgensen M, et al.. 2007. Respiration of Escherichia coli in the mouse intestine. Infect Immun 75:4891–4899. doi:10.1128/IAI.00484-07 17698572 PMC2044527

[40] Hagan EC, Lloyd AL, Rasko DA, Faerber GJ, Mobley HLT. 2010. Escherichia coli global gene expression in urine from women with urinary tract infection. PLoS Pathog 6:e1001187. doi:10.1371/journal.ppat.1001187 21085611 PMC2978726

[41] Shepherd M, Achard MES, Idris A, Totsika M, Phan M-D, Peters KM, Sarkar S, Ribeiro CA, Holyoake LV, Ladakis D, et al.. 2016. The cytochrome bd-I respiratory oxidase augments survival of multidrug-resistant Escherichia coli during infection. Sci Rep 6:35285. doi:10.1038/srep35285 27767067 PMC5073308

[42] Beebout CJ, Robertson GL, Reinfeld BI, Blee AM, Morales GH, Brannon JR, Chazin WJ, Rathmell WK, Rathmell JC, Gama V, et al.. 2022. Uropathogenic Escherichia coli subverts mitochondrial metabolism to enable intracellular bacterial pathogenesis in urinary tract infection. Nat Microbiol 7:1348–1360. doi:10.1038/s41564-022-01205-w 35995841 PMC9756876

[43] Xiao X, Song G, Lu H, Zheng W, Meng P, Peng W, Yang J, Wang M, Zhu J, Wang J, et al.. 2025. Cytochrome bd-II oxidase CyxA promotes the pathogenicity of Klebsiella pneumoniae by resisting oxidative stress. Virulence 16:2590244. doi:10.1080/21505594.2025.2590244 41236911 PMC12629338

[44] Stritzker J, Janda J, Schoen C, Taupp M, Pilgrim S, Gentschev I, Schreier P, Geginat G, Goebel W. 2004. Growth, virulence, and immunogenicity of Listeria monocytogenes aro mutants. Infect Immun 72:5622–5629. doi:10.1128/IAI.72.10.5622-5629.2004 15385459 PMC517589

[45] Corbett D, Goldrick M, Fernandes VE, Davidge K, Poole RK, Andrew PW, Cavet J, Roberts IS. 2017. Listeria monocytogenes has both cytochrome bd-type and cytochrome aa_3_-type terminal oxidases, which allow growth at different oxygen levels, and both are important in infection. Infect Immun 85:e00354-17. doi:10.1128/IAI.00354-17 28808161 PMC5649020

[46] Chen GY, McDougal CE, D’Antonio MA, Portman JL, Sauer J-D. 2017. A genetic screen reveals that synthesis of 1,4-dihydroxy-2-naphthoate (DHNA), but not full-length menaquinone, is required for Listeria monocytogenes cytosolic survival. mBio 8:e00119-17. doi:10.1128/mBio.00119-17 28325762 PMC5362031

[47] Rivera-Lugo R, Deng D, Anaya-Sanchez A, Tejedor-Sanz S, Tang E, Reyes Ruiz VM, Smith HB, Titov DV, Sauer J-D, Skaar EP, et al.. 2022. Listeria monocytogenes requires cellular respiration for NAD^+^ regeneration and pathogenesis. eLife 11:e75424. doi:10.7554/eLife.75424 35380108 PMC9094743

[48] Boot M, Jim KK, Liu T, Commandeur S, Lu P, Verboom T, Lill H, Bitter W, Bald D. 2017. A fluorescence-based reporter for monitoring expression of mycobacterial cytochrome bd in response to antibacterials and during infection. Sci Rep 7:10665. doi:10.1038/s41598-017-10944-4 28878275 PMC5587683

[49] Kana BD, Weinstein EA, Avarbock D, Dawes SS, Rubin H, Mizrahi V. 2001. Characterization of the cydAB-encoded cytochrome bd oxidase from Mycobacterium smegmatis. J Bacteriol 183:7076–7086. doi:10.1128/JB.183.24.7076-7086.2001 11717265 PMC95555

[50] Lu P, Heineke MH, Koul A, Andries K, Cook GM, Lill H, van Spanning R, Bald D. 2015. The cytochrome bd-type quinol oxidase is important for survival of Mycobacterium smegmatis under peroxide and antibiotic-induced stress. Sci Rep 5:10333. doi:10.1038/srep10333 26015371 PMC4450806

[51] Matsoso LG, Kana BD, Crellin PK, Lea-Smith DJ, Pelosi A, Powell D, Dawes SS, Rubin H, Coppel RL, Mizrahi V. 2005. Function of the cytochrome bc_1_-aa_3_ branch of the respiratory network in mycobacteria and network adaptation occurring in response to its disruption. J Bacteriol 187:6300–6308. doi:10.1128/JB.187.18.6300-6308.2005 16159762 PMC1236647

[52] Cai Y, Jaecklein E, Mackenzie JS, Papavinasasundaram K, Olive AJ, Chen X, Steyn AJC, Sassetti CM. 2021. Host immunity increases Mycobacterium tuberculosis reliance on cytochrome bd oxidase. PLoS Pathog 17:e1008911. doi:10.1371/journal.ppat.1008911 34320028 PMC8351954

[53] Shi L, Sohaskey CD, Kana BD, Dawes S, North RJ, Mizrahi V, Gennaro ML. 2005. Changes in energy metabolism of Mycobacterium tuberculosis in mouse lung and under in vitro conditions affecting aerobic respiration. Proc Natl Acad Sci USA 102:15629–15634. doi:10.1073/pnas.0507850102 16227431 PMC1255738

[54] Beites T, O’Brien K, Tiwari D, Engelhart CA, Walters S, Andrews J, Yang H-J, Sutphen ML, Weiner DM, Dayao EK, et al.. 2019. Plasticity of the Mycobacterium tuberculosis respiratory chain and its impact on tuberculosis drug development. Nat Commun 10:4970. doi:10.1038/s41467-019-12956-2 31672993 PMC6823465

[55] Kalia NP, Hasenoehrl EJ, Ab Rahman NB, Koh VH, Ang MLT, Sajorda DR, Hards K, Grüber G, Alonso S, Cook GM, et al.. 2017. Exploiting the synthetic lethality between terminal respiratory oxidases to kill Mycobacterium tuberculosis and clear host infection. Proc Natl Acad Sci USA 114:7426–7431. doi:10.1073/pnas.1706139114 28652330 PMC5514758

[56] Scherr N, Bieri R, Thomas SS, Chauffour A, Kalia NP, Schneide P, Ruf M-T, Lamelas A, Manimekalai MSS, Grüber G, et al.. 2018. Targeting the Mycobacterium ulcerans cytochrome bc_1_:aa_3_ for the treatment of Buruli ulcer. Nat Commun 9:5370. doi:10.1038/s41467-018-07804-8 30560872 PMC6299076

[57] Jo J, Cortez KL, Cornell WC, Price-Whelan A, Dietrich LE. 2017. An orphan cbb_3_-type cytochrome oxidase subunit supports Pseudomonas aeruginosa biofilm growth and virulence. eLife 6:e30205. doi:10.7554/eLife.30205 29160206 PMC5697931

[58] Narra HP, Alsing J, Sahni A, Montini M, Zafar Y, Sahni SK. 2023. A small non-coding RNA mediates transcript stability and expression of cytochrome bd ubiquinol oxidase subunit I in Rickettsia conorii. Int J Mol Sci 24:4008. doi:10.3390/ijms24044008 36835430 PMC9960880

[59] Shah DH. 2014. RNA sequencing reveals differences between the global transcriptomes of Salmonella enterica serovar Enteritidis strains with high and low pathogenicities. Appl Environ Microbiol 80:896–906. doi:10.1128/AEM.02740-13 24271167 PMC3911198

[60] Craig M, Sadik AY, Golubeva YA, Tidhar A, Slauch JM. 2013. Twin-arginine translocation system (tat) mutants of Salmonella are attenuated due to envelope defects, not respiratory defects. Mol Microbiol 89:887–902. doi:10.1111/mmi.12318 23822642 PMC3811912

[61] Jones-Carson J, Husain M, Liu L, Orlicky DJ, Vázquez-Torres A. 2016. Cytochrome bd-dependent bioenergetics and antinitrosative defenses in Salmonella pathogenesis. mBio 7:e02052-16. doi:10.1128/mBio.02052-16 PMC5181779 27999164

[62] Rivera-Chávez F, Zhang LF, Faber F, Lopez CA, Byndloss MX, Olsan EE, Xu G, Velazquez EM, Lebrilla CB, Winter SE, et al.. 2016. Depletion of butyrate-producing Clostridia from the gut microbiota drives an aerobic luminal expansion of Salmonella. Cell Host Microbe 19:443–454. doi:10.1016/j.chom.2016.03.004 27078066 PMC4832419

[63] Turner AK, Barber LZ, Wigley P, Muhammad S, Jones MA, Lovell MA, Hulme S, Barrow PA. 2003. Contribution of proton-translocating proteins to the virulence of Salmonella enterica serovars Typhimurium, Gallinarum, and Dublin in chickens and mice. Infect Immun 71:3392–3401. doi:10.1128/IAI.71.6.3392-3401.2003 12761123 PMC155768

[64] Barrow PA, Berchieri A, Freitas Neto O de, Lovell M. 2015. The contribution of aerobic and anaerobic respiration to intestinal colonization and virulence for Salmonella Typhimurium in the chicken. Avian Pathol 44:401–407. doi:10.1080/03079457.2015.1062841 26443064

[65] Silva-Valenzuela CA, Molina-Quiroz RC, Desai P, Valenzuela C, Porwollik S, Zhao M, Hoffman RM, Andrews-Polymenis H, Contreras I, Santiviago CA, et al.. 2015. Analysis of two complementary single-gene deletion mutant libraries of Salmonella Typhimurium in intraperitoneal infection of BALB/c mice. Front Microbiol 6:1455. doi:10.3389/fmicb.2015.01455 26779130 PMC4700939

[66] Way SS, Sallustio S, Magliozzo RS, Goldberg MB. 1999. Impact of either elevated or decreased levels of cytochrome bd expression on Shigella flexneri virulence. J Bacteriol 181:1229–1237. doi:10.1128/JB.181.4.1229-1237.1999 9973350 PMC93501

[67] Tinevez J-Y, Arena ET, Anderson M, Nigro G, Injarabian L, André A, Ferrari M, Campbell-Valois F-X, Devin A, Shorte SL, et al.. 2019. Shigella-mediated oxygen depletion is essential for intestinal mucosa colonization. Nat Microbiol 4:2001–2009. doi:10.1038/s41564-019-0525-3 31383999 PMC6817363

[68] Hammer ND, Reniere ML, Cassat JE, Zhang Y, Hirsch AO, Indriati Hood M, Skaar EP. 2013. Two heme-dependent terminal oxidases power Staphylococcus aureus organ-specific colonization of the vertebrate host. mBio 4:e00241-13. doi:10.1128/mBio.00241-13 23900169 PMC3735196

[69] Yamamoto Y, Poyart C, Trieu-Cuot P, Lamberet G, Gruss A, Gaudu P. 2005. Respiration metabolism of Group B Streptococcus is activated by environmental haem and quinone and contributes to virulence. Mol Microbiol 56:525–534. doi:10.1111/j.1365-2958.2005.04555.x 15813741

[70] Joubert L, Dagieu J-B, Fernandez A, Derré-Bobillot A, Borezée-Durant E, Fleurot I, Gruss A, Lechardeur D. 2017. Visualization of the role of host heme on the virulence of the heme auxotroph Streptococcus agalactiae. Sci Rep 7:40435. doi:10.1038/srep40435 28091535 PMC5238366

[71] Fu Y, Waldor MK, Mekalanos JJ. 2013. Tn-Seq analysis of Vibrio cholerae intestinal colonization reveals a role for T6SS-mediated antibacterial activity in the host. Cell Host Microbe 14:652–663. doi:10.1016/j.chom.2013.11.001 24331463 PMC3951154

[72] Van Alst AJ, DiRita VJ. 2020. Aerobic metabolism in Vibrio cholerae is required for population expansion during infection. mBio 11:e01989-20. doi:10.1128/mBio.01989-20 32873763 PMC7468205

[73] Van Alst AJ, Demey LM, DiRita VJ. 2022. Vibrio cholerae requires oxidative respiration through the bd-I and cbb_3_ oxidases for intestinal proliferation. PLOS Pathog 18:e1010102. doi:10.1371/journal.ppat.1010102 35500027 PMC9109917

[74] Murali R, Gennis RB, Hemp J. 2021. Evolution of the cytochrome bd oxygen reductase superfamily and the function of CydAA’ in Archaea. ISME J 15:3534–3548. doi:10.1038/s41396-021-01019-4 34145390 PMC8630170

[75] Abramson J, Riistama S, Larsson G, Jasaitis A, Svensson-Ek M, Laakkonen L, Puustinen A, Iwata S, Wikström M. 2000. The structure of the ubiquinol oxidase from Escherichia coli and its ubiquinone binding site. Nat Struct Biol 7:910–917. doi:10.1038/82824 11017202

[76] Belevich I, Borisov VB, Zhang J, Yang K, Konstantinov AA, Gennis RB, Verkhovsky MI. 2005. Time-resolved electrometric and optical studies on cytochrome bd suggest a mechanism of electron-proton coupling in the di-heme active site. Proc Natl Acad Sci USA 102:3657–3662. doi:10.1073/pnas.0405683102 15728392 PMC553295

[77] Siletsky SA, Borisov VB. 2021. Proton pumping and non-pumping terminal respiratory oxidases: active sites intermediates of these molecular machines and their derivatives. Int J Mol Sci 22:10852. doi:10.3390/ijms221910852 34639193 PMC8509429

[78] Al-Attar S, Yu Y, Pinkse M, Hoeser J, Friedrich T, Bald D, de Vries S. 2016. Cytochrome bd displays significant quinol peroxidase activity. Sci Rep 6:27631. doi:10.1038/srep27631 27279363 PMC4899803

[79] Borisov VB, Forte E, Siletsky SA, Sarti P, Giuffrè A. 2015. Cytochrome bd from Escherichia coli catalyzes peroxynitrite decomposition. Biochim Biophys Acta 1847:182–188. doi:10.1016/j.bbabio.2014.10.006 25449967

[80] Giuffrè A, Borisov VB, Arese M, Sarti P, Forte E. 2014. Cytochrome bd oxidase and bacterial tolerance to oxidative and nitrosative stress. Biochim Biophys Acta 1837:1178–1187. doi:10.1016/j.bbabio.2014.01.016 24486503

[81] Borisov VB, Forte E. 2021. Terminal oxidase cytochrome bd protects bacteria against hydrogen sulfide toxicity. Biochemistry (Mosc) 86:22–32. doi:10.1134/S000629792101003X 33705279

[82] Rice CW, Hempfling WP. 1978. Oxygen-limited continuous culture and respiratory energy conservation in Escherichia coli. J Bacteriol 134:115–124. doi:10.1128/jb.134.1.115-124.1978 25879 PMC222225

[83] Cotter PA, Chepuri V, Gennis RB, Gunsalus RP. 1990. Cytochrome o (cyoABCDE) and d (cydAB) oxidase gene expression in Escherichia coli is regulated by oxygen, pH, and the fnr gene product. J Bacteriol 172:6333–6338. doi:10.1128/jb.172.11.6333-6338.1990 2172211 PMC526817

[84] Avetisyan AV, Bogachev AV, Murtasina RA, Skulachev VP. 1992. Involvement of a d-type oxidase in the Na^+^-motive respiratory chain of Escherichia coli growing under low Δμ_H+_ conditions. FEBS Lett 306:199–202. doi:10.1016/0014-5793(92)80999-w 1321735

[85] Wall D, Delaney JM, Fayet O, Lipinska B, Yamamoto T, Georgopoulos C. 1992. arc-dependent thermal regulation and extragenic suppression of the Escherichia coli cytochrome d operon. J Bacteriol 174:6554–6562. doi:10.1128/jb.174.20.6554-6562.1992 1328158 PMC207623

[86] Bogachev AV, Murtazine RA, Shestopalov AI, Skulachev VP. 1995. Induction of the Escherichia coli cytochrome d by low Δμ_H+_ and by sodium ions. Eur J Biochem 232:304–308. doi:10.1111/j.1432-1033.1995.tb20812.x 7556165

[87] Ashcroft JR, Haddock BA. 1975. Synthesis of alternative membrane-bound redox carriers during aerobic growth of Escherichia coli in the presence of potassium cyanide. Biochem J 148:349–352. doi:10.1042/bj1480349 1098659 PMC1165546

[88] Borisov VB, Siletsky SA, Paiardini A, Hoogewijs D, Forte E, Giuffrè A, Poole RK. 2021. Bacterial oxidases of the cytochrome bd family: redox enzymes of unique structure, function, and utility as drug targets. Antioxid Redox Signal 34:1280–1318. doi:10.1089/ars.2020.8039 32924537 PMC8112716

[89] Friedrich T, Wohlwend D, Borisov VB. 2022. Recent advances in structural studies of cytochrome bd and its potential application as a drug target. Int J Mol Sci 23:3166. doi:10.3390/ijms23063166 35328590 PMC8951039

[90] World Health Organization. 2024. Global tuberculosis report 2024

[91] Gomez JE, McKinney JD. 2004. M. tuberculosis persistence, latency, and drug tolerance. Tuberculosis (Edinb) 84:29–44. doi:10.1016/j.tube.2003.08.003 14670344

[92] Dhar N, McKinney JD. 2007. Microbial phenotypic heterogeneity and antibiotic tolerance. Curr Opin Microbiol 10:30–38. doi:10.1016/j.mib.2006.12.007 17215163

[93] Nathan C. 2012. Fresh approaches to anti-infective therapies. Sci Transl Med 4:140sr2. doi:10.1126/scitranslmed.3003081 22745440 PMC3712344

[94] Ehrt S, Schnappinger D, Rhee KY. 2018. Metabolic principles of persistence and pathogenicity in Mycobacterium tuberculosis. Nat Rev Microbiol 16:496–507. doi:10.1038/s41579-018-0013-4 29691481 PMC6045436

[95] Cronan MR. 2022. In the thick of it: formation of the tuberculous granuloma and its effects on host and therapeutic responses. Front Immunol 13:820134. doi:10.3389/fimmu.2022.820134 35320930 PMC8934850

[96] Sotgiu G, Centis R, D’ambrosio L, Migliori GB. 2015. Tuberculosis treatment and drug regimens. Cold Spring Harb Perspect Med 5:a017822. doi:10.1101/cshperspect.a017822 25573773 PMC4448591

[97] Borisov S, Danila E, Maryandyshev A, Dalcolmo M, Miliauskas S, Kuksa L, Manga S, Skrahina A, Diktanas S, Codecasa LR, et al.. 2019. Surveillance of adverse events in the treatment of drug-resistant tuberculosis: first global report. Eur Respir J 54:1901522. doi:10.1183/13993003.01522-2019 31601711

[98] Akkerman O, Aleksa A, Alffenaar J-W, Al-Marzouqi NH, Arias-Guillén M, Belilovski E, Bernal E, Boeree MJ, Borisov SE, Bruchfeld J, et al.. 2019. Surveillance of adverse events in the treatment of drug-resistant tuberculosis: a global feasibility study. Int J Infect Dis 83:72–76. doi:10.1016/j.ijid.2019.03.036 30953827

[99] Andries K, Verhasselt P, Guillemont J, Göhlmann HWH, Neefs J-M, Winkler H, Van Gestel J, Timmerman P, Zhu M, Lee E, et al.. 2005. A diarylquinoline drug active on the ATP synthase of Mycobacterium tuberculosis. Science 307:223–227. doi:10.1126/science.1106753 15591164

[100] Janssen Therapeutics, Division of Janssen Products, LP. 2019. Sirturo: highlights of prescribing information. Available from: https://www.accessdata.fda.gov/drugsatfda_docs/label/2024/204384s019lbl.pdf. Retrieved 26 Nov 2024.

[101] Rao SPS, Alonso S, Rand L, Dick T, Pethe K. 2008. The protonmotive force is required for maintaining ATP homeostasis and viability of hypoxic, nonreplicating Mycobacterium tuberculosis. Proc Natl Acad Sci USA 105:11945–11950. doi:10.1073/pnas.0711697105 18697942 PMC2575262

[102] Gengenbacher M, Rao SPS, Pethe K, Dick T. 2010. Nutrient-starved, non-replicating Mycobacterium tuberculosis requires respiration, ATP synthase and isocitrate lyase for maintenance of ATP homeostasis and viability. Microbiology (Reading) 156:81–87. doi:10.1099/mic.0.033084-0 19797356

[103] Hards K, Robson JR, Berney M, Shaw L, Bald D, Koul A, Andries K, Cook GM. 2015. Bactericidal mode of action of bedaquiline. J Antimicrob Chemother 70:2028–2037. doi:10.1093/jac/dkv054 25754998

[104] Foo C-Y, Pethe K, Lupien A. 2020. Oxidative phosphorylation—an update on a new, essential target space for drug discovery in Mycobacterium tuberculosis. Appl Sci (Basel) 10:2339. doi:10.3390/app10072339

[105] Hasenoehrl EJ, Wiggins TJ, Berney M. 2020. Bioenergetic inhibitors: antibiotic efficacy and mechanisms of action in Mycobacterium tuberculosis. Front Cell Infect Microbiol 10:611683. doi:10.3389/fcimb.2020.611683 33505923 PMC7831573

[106] Cook GM, Hards K, Vilchèze C, Hartman T, Berney M. 2014. Energetics of respiration and oxidative phosphorylation in mycobacteria, p 389–409. *In* Molecular genetics of mycobacteria. John Wiley & Sons, Ltd. 10.1128/microbiolspec.MGM2-0015-2013 PMC4205543 25346874

[107] Small JL, Park SW, Kana BD, Ioerger TR, Sacchettini JC, Ehrt S. 2013. Perturbation of cytochrome c maturation reveals adaptability of the respiratory chain in Mycobacterium tuberculosis. mBio 4:e00475-13. doi:10.1128/mBio.00475-13 24045640 PMC3781833

[108] Arora K, Ochoa-Montaño B, Tsang PS, Blundell TL, Dawes SS, Mizrahi V, Bayliss T, Mackenzie CJ, Cleghorn LAT, Ray PC, et al.. 2014. Respiratory flexibility in response to inhibition of cytochrome C oxidase in Mycobacterium tuberculosis. Antimicrob Agents Chemother 58:6962–6965. doi:10.1128/AAC.03486-14 25155596 PMC4249445

[109] Foo CS, Lupien A, Kienle M, Vocat A, Benjak A, Sommer R, Lamprecht DA, Steyn AJC, Pethe K, Piton J, et al.. 2018. Arylvinylpiperazine amides, a new class of potent inhibitors targeting QcrB of Mycobacterium tuberculosis. mBio 9:e01276-18. doi:10.1128/mBio.01276-18 30301850 PMC6178619

[110] Pethe K, Bifani P, Jang J, Kang S, Park S, Ahn S, Jiricek J, Jung J, Jeon HK, Cechetto J, et al.. 2013. Discovery of Q203, a potent clinical candidate for the treatment of tuberculosis. Nat Med 19:1157–1160. doi:10.1038/nm.3262 23913123

[111] Kim J, Choi J, Kang H, Ahn J, Hutchings J, van Niekerk C, Park D, Kim J, Jeon Y, Nam K, et al.. 2022. Safety, tolerability, and pharmacokinetics of telacebec (Q203), a new antituberculosis agent, in healthy subjects. Antimicrob Agents Chemother 66:e0143621. doi:10.1128/AAC.01436-21 34694872 PMC8765288

[112] Lamprecht DA, Finin PM, Rahman M, Cumming BM, Russell SL, Jonnala SR, Adamson JH, Steyn AJC. 2016. Turning the respiratory flexibility of Mycobacterium tuberculosis against itself. Nat Commun 7:12393. doi:10.1038/ncomms12393 27506290 PMC4987515

[113] Almeida DV, Converse PJ, Omansen TF, Tyagi S, Tasneen R, Kim J, Nuermberger EL. 2020. Telacebec for ultrashort treatment of Buruli ulcer in a mouse model. Antimicrob Agents Chemother 64:e00259-20. doi:10.1128/AAC.00259-20 32205344 PMC7269501

[114] Foxman B. 2014. Urinary tract infection syndromes: occurrence, recurrence, bacteriology, risk factors, and disease burden. Infect Dis Clin North Am 28:1–13. doi:10.1016/j.idc.2013.09.003 24484571

[115] Klevens RM, Edwards JR, Richards CL Jr, Horan TC, Gaynes RP, Pollock DA, Cardo DM. 2007. Estimating health care-associated infections and deaths in U.S. hospitals, 2002. Public Health Rep 122:160–166. doi:10.1177/003335490712200205 17357358 PMC1820440

[116] Scott RD. 2009. The direct medical costs of healthcare-associated infections in US hospitals and the benefits of prevention. Publication CS200891-A. Centers for Disease Control and Prevention.

[117] Yang X, Chen H, Zheng Y, Qu S, Wang H, Yi F. 2022. Disease burden and long-term trends of urinary tract infections: a worldwide report. Front Public Health 10. doi:10.3389/fpubh.2022.888205 PMC9363895 35968451

[118] Iacovelli V, Gaziev G, Topazio L, Bove P, Vespasiani G, Finazzi Agrò E. 2014. Nosocomial urinary tract infections: a review. Urologia 81:222–227. doi:10.5301/uro.5000092 25451882

[119] Foxman B. 2010. The epidemiology of urinary tract infection. Nat Rev Urol 7:653–660. doi:10.1038/nrurol.2010.190 21139641

[120] Kennedy EH, Greene MT, Saint S. 2013. Estimating hospital costs of catheter-associated urinary tract infection. J Hosp Med 8:519–522. doi:10.1002/jhm.2079 24038833 PMC3786530

[121] Raeispour M, Ranjbar R. 2018. Antibiotic resistance, virulence factors and genotyping of Uropathogenic Escherichia coli strains. Antimicrob Resist Infect Control 7:118. doi:10.1186/s13756-018-0411-4 30305891 PMC6171155

[122] Wang ZJ, Joe BN, Coakley FV, Zaharchuk G, Busse R, Yeh BM. 2008. Urinary oxygen tension measurement in humans using magnetic resonance imaging. Acad Radiol 15:1467–1473. doi:10.1016/j.acra.2008.04.013 18995198 PMC2605795

[123] Conover MS, Hadjifrangiskou M, Palermo JJ, Hibbing ME, Dodson KW, Hultgren SJ. 2016. Metabolic requirements of Escherichia coli in intracellular bacterial communities during urinary tract infection pathogenesis. mBio 7:e00104-16. doi:10.1128/mBio.00104-16 27073089 PMC4959519

[124] Floyd KA, Mitchell CA, Eberly AR, Colling SJ, Zhang EW, DePas W, Chapman MR, Conover M, Rogers BR, Hultgren SJ, et al.. 2016. The ubiI (VisC) aerobic ubiquinone synthase is required for expression of type 1 pili, biofilm formation, and pathogenesis in uropathogenic Escherichia coli. J Bacteriol 198:2662–2672. doi:10.1128/JB.00030-16 27161114 PMC5019064

[125] Eberly AR, Floyd KA, Beebout CJ, Colling SJ, Fitzgerald MJ, Stratton CW, Schmitz JE, Hadjifrangiskou M. 2017. Biofilm formation by uropathogenic Escherichia coli is favored under oxygen conditions that mimic the bladder environment. Int J Mol Sci 18:2077. doi:10.3390/ijms18102077 28973965 PMC5666759

[126] Kranz RG, Barassi CA, Gennis RB. 1984. Immunological analysis of the heme proteins present in aerobically grown Escherichia coli. J Bacteriol 158:1191–1194. doi:10.1128/jb.158.3.1191-1194.1984 6373739 PMC215574

[127] Beebout CJ, Eberly AR, Werby SH, Reasoner SA, Brannon JR, De S, Fitzgerald MJ, Huggins MM, Clayton DB, Cegelski L, et al.. 2019. Respiratory heterogeneity shapes biofilm formation and host colonization in uropathogenic Escherichia coli. mBio 10:e02400-18. doi:10.1128/mBio.02400-18 30940709 PMC6445943

[128] Mogi T, Akimoto S, Endou S, Watanabe-Nakayama T, Mizuochi-Asai E, Miyoshi H. 2006. Probing the ubiquinol-binding site in cytochrome bd by site-directed mutagenesis. Biochemistry 45:7924–7930. doi:10.1021/bi060192w 16784245

[129] Safarian S, Hahn A, Mills DJ, Radloff M, Eisinger ML, Nikolaev A, Meier-Credo J, Melin F, Miyoshi H, Gennis RB, et al.. 2019. Active site rearrangement and structural divergence in prokaryotic respiratory oxidases. Science 366:100–104. doi:10.1126/science.aay0967 31604309

[130] Tiku V, Tan M-W, Dikic I. 2020. Mitochondrial functions in infection and immunity. Trends Cell Biol 30:263–275. doi:10.1016/j.tcb.2020.01.006 32200805 PMC7126537

[131] Borisov VB, Verkhovsky MI. 2015. Oxygen as acceptor. EcoSal Plus 6. doi:10.1128/ecosalplus.ESP-0012-2015 PMC11575855 26734697

[132] Krab K, Kempe H, Wikström M. 2011. Explaining the enigmatic K_M_ for oxygen in cytochrome c oxidase: a kinetic model. Biochim Biophys Acta 1807:348–358. doi:10.1016/j.bbabio.2010.12.015 21211514

[133] Fulda S, Debatin K-M. 2007. HIF-1-regulated glucose metabolism: a key to apoptosis resistance? Cell Cycle 6:790–792. doi:10.4161/cc.6.7.4084 17404504

[134] World Health Organization. 2024. Cholera, 2023, p 481–495. *In* Weekly epidemiological record. Vol. 99.

[135] Global Task Force on Cholera Control. 2017. Ending cholera: a global roadmap to 2030.

[136] Baker-Austin C, Trinanes J, Gonzalez-Escalona N, Martinez-Urtaza J. 2017. Non-cholera vibrios: the microbial barometer of climate change. Trends Microbiol 25:76–84. doi:10.1016/j.tim.2016.09.008 27843109

[137] Merrell DS, Butler SM, Qadri F, Dolganov NA, Alam A, Cohen MB, Calderwood SB, Schoolnik GK, Camilli A. 2002. Host-induced epidemic spread of the cholera bacterium. Nature 417:642–645. doi:10.1038/nature00778 12050664 PMC2776822

[138] Hartley DM, Morris JG Jr, Smith DL. 2006. Hyperinfectivity: a critical element in the ability of V. cholerae to cause epidemics? PLOS Med 3:e7. doi:10.1371/journal.pmed.0030007 16318414 PMC1298942

[139] Harris JB, LaRocque RC, Chowdhury F, Khan AI, Logvinenko T, Faruque ASG, Ryan ET, Qadri F, Calderwood SB. 2008. Susceptibility to Vibrio cholerae infection in a cohort of household contacts of patients with cholera in Bangladesh. PLoS Negl Trop Dis 2:e221. doi:10.1371/journal.pntd.0000221 18398491 PMC2271133

[140] Weil AA, Khan AI, Chowdhury F, LaRocque RC, Faruque ASG, Ryan ET, Calderwood SB, Qadri F, Harris JB. 2009. Clinical outcomes in household contacts of patients with cholera in Bangladesh. Clin Infect Dis 49:1473–1479. doi:10.1086/644779 19842974 PMC2783773

[141] Pezzoli L. 2020. Global oral cholera vaccine use, 2013–2018. Vaccine (Auckl) 38:A132–A140. doi:10.1016/j.vaccine.2019.08.086 PMC10967685 31519444

[142] Davies HG, Bowman C, Luby SP. 2017. Cholera – management and prevention. J Infect 74:S66–S73. doi:10.1016/S0163-4453(17)30194-9 28646965

[143] Pietroni MAC. 2020. Case management of cholera. Vaccine (Auckl) 38:A105–A109. doi:10.1016/j.vaccine.2019.09.098 31668817

[144] Sousa FBM, Nolêto IRSG, Chaves LS, Pacheco G, Oliveira AP, Fonseca MMV, Medeiros JVR. 2020. A comprehensive review of therapeutic approaches available for the treatment of cholera. J Pharm Pharmacol 72:1715–1731. doi:10.1111/jphp.13344 32737883

[145] Shapiro BJ, Levade I, Kovacikova G, Taylor RK, Almagro-Moreno S. 2016. Origins of pandemic Vibrio cholerae from environmental gene pools. Nat Microbiol 2:16240. doi:10.1038/nmicrobiol.2016.240 27991885

[146] Clemens JD, Nair GB, Ahmed T, Qadri F, Holmgren J. 2017. Cholera. Lancet 390:1539–1549. doi:10.1016/S0140-6736(17)30559-7 28302312

[147] Kanungo S, Azman AS, Ramamurthy T, Deen J, Dutta S. 2022. Cholera. Lancet 399:1429–1440. doi:10.1016/S0140-6736(22)00330-0 35397865

[148] Ramamurthy T, Pragasam AK, Taylor-Brown A, Will RC, Vasudevan K, Das B, Srivastava SK, Chowdhury G, Mukhopadhyay AK, Dutta S, et al.. 2022. Vibrio cholerae O139 genomes provide a clue to why it may have failed to usher in the eighth cholera pandemic. Nat Commun 13:3864. doi:10.1038/s41467-022-31391-4 35790755 PMC9256687

[149] Chun J, Grim CJ, Hasan NA, Lee JH, Choi SY, Haley BJ, Taviani E, Jeon Y-S, Kim DW, Lee J-H, et al.. 2009. Comparative genomics reveals mechanism for short-term and long-term clonal transitions in pandemic Vibrio cholerae. Proc Natl Acad Sci USA 106:15442–15447. doi:10.1073/pnas.0907787106 19720995 PMC2741270

[150] Boucher Y. 2016. Sustained local diversity of Vibrio cholerae O1 biotypes in a previously cholera-free country. mBio 7:e00570-16. doi:10.1128/mBio.00570-16 27143391 PMC4959653

[151] Balasubramanian D, López-Pérez M, Almagro-Moreno S. 2023. Cholera dynamics and the emergence of pandemic *Vibrio cholerae*, p 127–147. *In* Almagro-Moreno S, Pukatzki S (ed), Vibrio spp. infections. Springer International Publishing, Cham. 10.1007/978-3-031-22997-8_7 36792874

[152] Zheng L, Kelly CJ, Colgan SP. 2015. Physiologic hypoxia and oxygen homeostasis in the healthy intestine. A review in the theme: cellular responses to hypoxia. Am J Physiol Cell Physiol 309:C350–C360. doi:10.1152/ajpcell.00191.2015 26179603 PMC4572369

[153] Millet YA, Alvarez D, Ringgaard S, von Andrian UH, Davis BM, Waldor MK. 2014. Insights into Vibrio cholerae intestinal colonization from monitoring fluorescently labeled bacteria. PLOS Pathog 10:e1004405. doi:10.1371/journal.ppat.1004405 25275396 PMC4183697

[154] Carreau A, El Hafny-Rahbi B, Matejuk A, Grillon C, Kieda C. 2011. Why is the partial oxygen pressure of human tissues a crucial parameter? small molecules and hypoxia. J Cell Mol Med 15:1239–1253. doi:10.1111/j.1582-4934.2011.01258.x 21251211 PMC4373326

[155] Hughes ER, Winter MG, Duerkop BA, Spiga L, Furtado de Carvalho T, Zhu W, Gillis CC, Büttner L, Smoot MP, Behrendt CL, et al.. 2017. Microbial respiration and formate oxidation as metabolic signatures of inflammation-associated dysbiosis. Cell Host Microbe 21:208–219. doi:10.1016/j.chom.2017.01.005 28182951 PMC5313043

[156] Heidelberg JF, Eisen JA, Nelson WC, Clayton RA, Gwinn ML, Dodson RJ, Haft DH, Hickey EK, Peterson JD, Umayam L, et al.. 2000. DNA sequence of both chromosomes of the cholera pathogen Vibrio cholerae. Nature 406:477–483. doi:10.1038/35020000 10952301 PMC8288016

[157] Bueno E, Sit B, Waldor MK, Cava F. 2018. Anaerobic nitrate reduction divergently governs population expansion of the enteropathogen Vibrio cholerae. Nat Microbiol 3:1346–1353. doi:10.1038/s41564-018-0253-0 30275512 PMC6443258

[158] Jones SA, Gibson T, Maltby RC, Chowdhury FZ, Stewart V, Cohen PS, Conway T. 2011. Anaerobic respiration of Escherichia coli in the mouse intestine. Infect Immun 79:4218–4226. doi:10.1128/IAI.05395-11 21825069 PMC3187261

[159] Lee K-M, Park Y, Bari W, Yoon MY, Go J, Kim SC, Lee H, Yoon SS. 2012. Activation of cholera toxin production by anaerobic respiration of trimethylamine N-oxide in Vibrio cholerae. J Biol Chem 287:39742–39752. doi:10.1074/jbc.M112.394932 23019319 PMC3501055

[160] Braun M, Thöny-Meyer L. 2005. Cytochrome c maturation and the physiological role of c-type cytochromes in Vibrio cholerae. J Bacteriol 187:5996–6004. doi:10.1128/JB.187.17.5996-6004.2005 16109941 PMC1196146

[161] CDC. 2024. Symptoms of whooping cough. Pertussis (Whooping Cough). Available from: https://www.cdc.gov/pertussis/signs-symptoms/index.html. Retrieved 24 Jun 2024.

[162] Plotkin SA. 2014. The pertussis problem. Clin Infect Dis 58:830–833. doi:10.1093/cid/cit934 24363332

[163] Cody CL, Baraff LJ, Cherry JD, Marcy SM, Manclark CR. 1981. Nature and rates of adverse reactions associated with DTP and DT immunizations in infants and children. Pediatrics 68:650–660. doi:10.1542/peds.68.5.650 7031583

[164] Kilgore PE, Salim AM, Zervos MJ, Schmitt H-J. 2016. Pertussis: microbiology, disease, treatment, and prevention. Clin Microbiol Rev 29:449–486. doi:10.1128/CMR.00083-15 27029594 PMC4861987

[165] CDC. 2024. Pertussis surveillance and trends. Whooping Cough (Pertussis). Available from: https://www.cdc.gov/pertussis/php/surveillance/index.html. Retrieved 24 Jun 2024.

[166] Warfel JM, Edwards KM. 2015. Pertussis vaccines and the challenge of inducing durable immunity. Curr Opin Immunol 35:48–54. doi:10.1016/j.coi.2015.05.008 26091979

[167] Witt MA, Katz PH, Witt DJ. 2012. Unexpectedly limited durability of immunity following acellular pertussis vaccination in preadolescents in a North American outbreak. Clin Infect Dis 54:1730–1735. doi:10.1093/cid/cis287 22423127

[168] Edwards KM, Berbers GAM. 2014. Immune responses to pertussis vaccines and disease. J Infect Dis 209:S10–S15. doi:10.1093/infdis/jit560 24158958

[169] Allen AC, Mills KHG. 2014. Improved pertussis vaccines based on adjuvants that induce cell-mediated immunity. Expert Rev Vaccines 13:1253–1264. doi:10.1586/14760584.2014.936391 25017925

[170] Warfel JM, Zimmerman LI, Merkel TJ. 2014. Acellular pertussis vaccines protect against disease but fail to prevent infection and transmission in a nonhuman primate model. Proc Natl Acad Sci USA 111:787–792. doi:10.1073/pnas.1314688110 24277828 PMC3896208

[171] Badhai J, Das SK. 2021. Genomic plasticity and antibody response of Bordetella bronchiseptica strain HT200, a natural variant from a thermal spring. FEMS Microbiol Lett 368:fnab035. doi:10.1093/femsle/fnab035 33856450

[172] Inatsuka CS, Julio SM, Cotter PA. 2005. Bordetella filamentous hemagglutinin plays a critical role in immunomodulation, suggesting a mechanism for host specificity. Proc Natl Acad Sci USA 102:18578–18583. doi:10.1073/pnas.0507910102 16339899 PMC1317942

[173] Henderson MW, Inatsuka CS, Sheets AJ, Williams CL, Benaron DJ, Donato GM, Gray MC, Hewlett EL, Cotter PA. 2012. Contribution of Bordetella filamentous hemagglutinin and adenylate cyclase toxin to suppression and evasion of interleukin-17-mediated inflammation. Infect Immun 80:2061–2075. doi:10.1128/IAI.00148-12 22473603 PMC3370597

[174] Julio SM, Inatsuka CS, Mazar J, Dieterich C, Relman DA, Cotter PA. 2009. Natural-host animal models indicate functional interchangeability between the filamentous haemagglutinins of Bordetella pertussis and Bordetella bronchiseptica and reveal a role for the mature C-terminal domain, but not the RGD motif, during infection. Mol Microbiol 71:1574–1590. doi:10.1111/j.1365-2958.2009.06623.x 19220744 PMC3422645

[175] Diavatopoulos DA, Cummings CA, Schouls LM, Brinig MM, Relman DA, Mooi FR. 2005. Bordetella pertussis, the causative agent of whooping cough, evolved from a distinct, human-associated lineage of B. bronchiseptica. PLoS Pathog 1:e45. doi:10.1371/journal.ppat.0010045 16389302 PMC1323478

[176] Parkhill J, Sebaihia M, Preston A, Murphy LD, Thomson N, Harris DE, Holden MTG, Churcher CM, Bentley SD, Mungall KL, et al.. 2003. Comparative analysis of the genome sequences of Bordetella pertussis, Bordetella parapertussis and Bordetella bronchiseptica. Nat Genet 35:32–40. doi:10.1038/ng1227 12910271

[177] Tuomanen EI, Hendley JO. 1983. Adherence of Bordetella pertussis to human respiratory epithelial cells. J Infect Dis 148:125–130. doi:10.1093/infdis/148.1.125 6309991

[178] Centers for Disease Control and Prevention (U.S). 2019. Antibiotic resistance threats in the United States, 2019. Centers for Disease Control and Prevention (U.S.).

[179] Miller WR, Arias CA. 2024. ESKAPE pathogens: antimicrobial resistance, epidemiology, clinical impact and therapeutics. Nat Rev Microbiol 22:598–616. doi:10.1038/s41579-024-01054-w 38831030 PMC13147291

